# Predicting ecosystem changes by a new model of ecosystem evolution

**DOI:** 10.1038/s41598-023-42529-9

**Published:** 2023-09-16

**Authors:** Katsuhiko Yoshida, Kenji Hata, Kazuto Kawakami, Syuntaro Hiradate, Takeshi Osawa, Naoki Kachi

**Affiliations:** 1 Biodiversity Division, National Institute for Environmental Studies, 16-2 Onogawa, Tsukuba, Ibaraki 305-8506 Japan; 2 College of Commerce, Nihon University, 5-2-1 Kinuta, Setagaya, Tokyo, 157-8570 Japan; 3 Department of Biological Sciences, Graduate School of Science, Tokyo Metropolitan University, 1-1 Minami-Osawa, Hachioji, Tokyo 192-0397 Japan; 4 Forestry and Forest Products Research Institute, 1 Matsunosato, Tsukuba, Ibaraki 305-8687 Japan; 5 Division of Bioproduction Environmental Sciences, Department of Agro-environmental Sciences, Faculty of Agriculture, Kyushu University, 744 Moto-Oka, Nishi-ku, Fukuoka, 819-0395 Japan; 6 Department of Tourism Science, Graduate School of Urban Environmental Sciences, Tokyo Metropolitan University, 1-1 Minami-Osawa, Hachioji, Tokyo 192-0397 Japan

**Keywords:** Community ecology, Conservation biology, Ecological modelling, Evolutionary ecology, Invasive species, Restoration ecology, Theoretical ecology, Ecology, Biodiversity, Community ecology, Conservation biology, Ecological modelling, Evolutionary ecology, Invasive species, Restoration ecology, Theoretical ecology

## Abstract

In recent years, computer simulation has been increasingly used to predict changes in actual ecosystems. In these studies, snapshots of ecosystems at certain points in time were instantly constructed without considering their evolutionary histories. However, it may not be possible to correctly predict future events unless their evolutionary processes are considered. In this study, we developed a new ecosystem model for reproducing the evolutionary process on an oceanic island, targeting Nakoudojima Island of the Ogasawara Islands. This model successfully reproduced the primitive ecosystem (the entire island covered with forest) prior to the invasion of alien species. Also, by adding multiple alien species to this ecosystem, we were able to reproduce temporal changes in the ecosystem of Nakoudojima Island after invasion of alien species. Then, we performed simulations in which feral goats were eradicated, as had actually been done on the island; these suggested that after the eradication of feral goats, forests were unlikely to be restored. In the ecosystems in which forests were not restored, arboreous plants with a high growth rate colonized during the early stage of evolution. As arboreous plants with a high growth rate consume a large amount of nutrient in soil, creating an oligotrophic state. As a result, plants cannot grow, and animal species that rely on plants cannot maintain their biomass. Consequently, many animals and plants become extinct as they cannot endure disturbances by alien species, and the ecosystem loses its resilience. Therefore, even if feral goats are eradicated, forests are not restored. Thus, the founder effect from the distant past influences future ecosystem changes. Our findings show that it is useful to consider the evolutionary process of an ecosystem in predicting its future events.

## Introduction

Computer simulation has been widely used to predict future events. In recent years, it has also been increasingly used to predict changes in actual ecosystems^1–9^. In these studies, snapshots of ecosystems at certain points in time were constructed without considering their evolutionary histories. However, in the case of ecosystems with a unique evolutionary history, such as oceanic islands, it may not be possible to correctly predict future events unless their evolutionary processes are considered. Therefore, in this study, we attempted to construct a model that reproduces the evolution of the oceanic island system for Nakoudojima Island in the Ogasawara Islands, a World Heritage Site. Using this model, we demonstrate that it can reproduce a distant past state (the original state) of the ecosystem and that it also can correctly reproduce temporal changes in the ecosystem of Nakoudojima Island after invasion of alien species to demonstrate a model’s ability to reproduce the real island. Then, we predict the future changes in the ecosystem of Nakoudojima Island.

The Ogasawara Islands are a group of oceanic islands located approximately 1000 km south of Tokyo (Fig. A.1). Nakoudojima Island, the subject of this study, is uninhabited, has an area of 1.4 km^2^ and is located approximately 50 km north of Chichijima Island. Prior to human settlement, all of the Ogasawara Islands were covered with forest^10–13^. Among the islands, Minamiioujima Island, which has never been settled by humans, is still almost entirely covered with forest^14^. Full-fledged human settlement in the islands began at the end of the nineteenth century, as a result of which goats, rats, white popinac (*Leucaena leucocephala*), and other non-native species were introduced to the islands. Subsequently, Nakoudojima Island became uninhabited after the government forced its residents to evacuate at the end of the Second World War, and the vegetation of the island greatly declined due to the influence of feral animals such as goats and rats^11,12,15,16^. The vegetation ratio in 1991 was as follows (places where plants cannot grow, such as rocky tracts, are excluded): forests 12.1%, grassland 64.5%, and denuded land 23.9%^17^. The efforts to eradicate feral goats, which had strongly influenced the decline of the local vegetation, began in 1997 and successfully ended in 1999^13,15,18^. At that time, it was expected that the island’s vegetation would recover; however, after more than 20 years, forests have not been restored, and denuded ground remains unvegetated^17,19–22^. There are some reported cases where vegetation was not restored after the eradication of feral goats^23–25^, which unfortunately currently includes the case of Nakoudojima Island.

**Figure 1:**
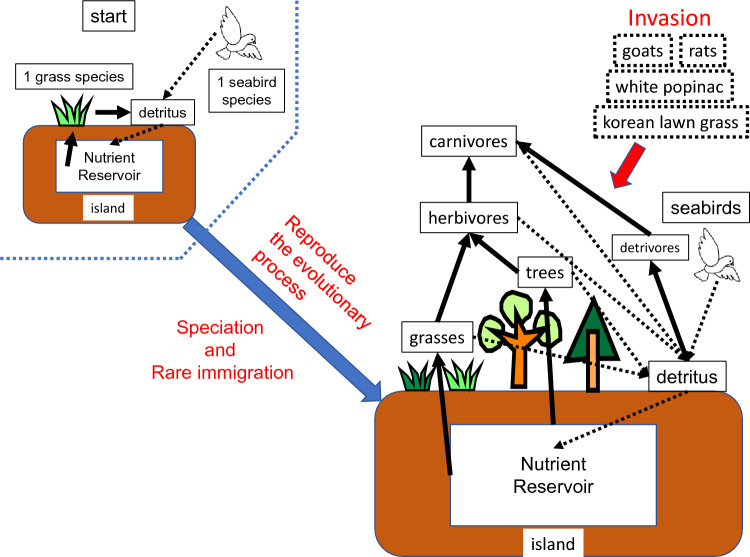
Schematic figure of the evolution and nutrient cycle of the model ecosystem. The simulation of the model ecosystem starts with one seabird species and one herbaceous plant species. Through frequent speciation within the ecosystem and rare species immigrations, the ecosystem transforms into a more diverse and complex structure. See Appendix 1 for the nutrient cycle process within the ecosystem, and Appendix 2 for details of the evolution process.

In this study, to determine why forest is not restored after goat eradication, we developed a new ecosystem evolution model, by incorporating evolutionary processes of an oceanic island ecosystem (Fig. 1, Appendix 2) into the model of Yoshida et al.^8^, which reproduces the nutrient cycle in an oceanic island ecosystem and successfully reproduced the ecosystem of Nakoudojima Island with high precision (see “Method” and Appendix 1). The novelty of the model is reproducing the evolution of the nutrient cycle process in an oceanic island ecosystem. There are almost no models that reproduce the evolutionary process while treating the nutrient circulation process in the ecosystem in detail. In this model, species consist of a single population, and changes in biomass of each species are calculated (using the unit of ton; when we compare the results of simulations with observed data, the amount of biomass is converted to other types of units, for example, the number of individuals). In this model ecosystem, plants (herbaceous and arboreous) absorb nutrient supplied from seabirds and grow. Ecological traits of individual plant species are set by applying the concept of Grime’s triangle^26–28^. To avoid giving advantages to particular species, all plant species are given 100 points, which are allocated across the three strategies: competitive (C), stress-tolerant (S), and ruderal (R). Ecological traits of plant species are determined based on these values (for details, see section 1-1 in Appendix 1). Plant species compete with each other for habitat space. Plants are ingested by herbivorous invertebrates, and herbivores are fed by carnivorous invertebrates (grazing food chain). Predator–prey interactions are set based on the Niche Model^29^. Changes in biomass of species are calculated using a Lotka–Volterra system with Holling type III functional response^30^. We consider three invasive species: feral goats, rats, and white popinac. Goats are generalist herbivores and interfere with seabirds’ nesting activity^31–33^ (Fig. 1). Rats are generalist omnivores and also scavengers. White popinac is a pioneer species and can fix nitrogen^34^. The model incorporates the detritus food chain and the process of detritus decomposition. A part of the detritus produced by plants and animals (droppings, carcasses, and litter) is consumed by detritivores, and another part of it is removed from the system through weathering. The remainder, together with the droppings of detritivores themselves, is decomposed into nutrient, which are available to plants again, and stored in the underground reservoir. We image one time step in the model as one day. Then, values of the metabolism efficiency of animal species per day are determined based on Yodzis and Innes^35^ (section 2 in Appendix 1). However, we cannot set values per day for all parameters. Then, in this paper, we show results of simulations by using “time step” as a unit of time.

**Figure 2:**
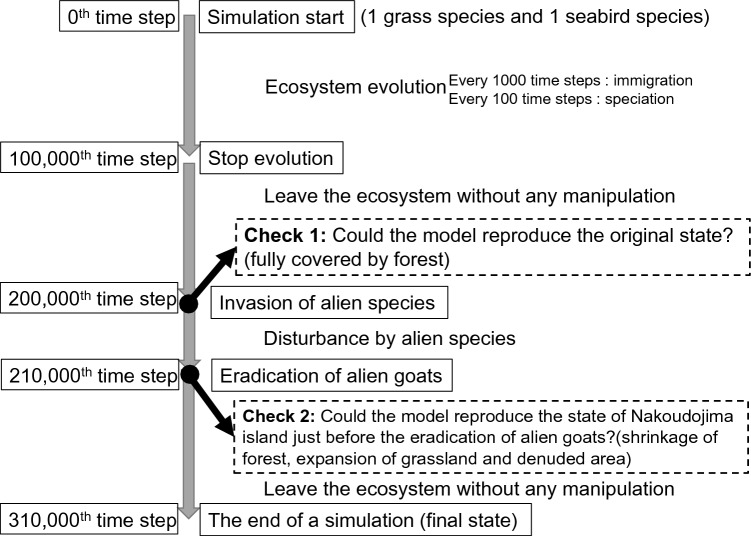
Schematic diagram of procedure of a simulation. Immigration: a completely new species immigrates to the ecosystem. Speciation: one species is selected from the constituent species of the ecosystem and given an opportunity for speciation. A subpopulation of the species is separated from the main population of the species and becomes a new species.

A complex ecosystem like this does not occur overnight. Although no animals and plants initially exist on an oceanic island that has appeared far away from other land, the biodiversity on the island increases through rare species immigrations and their repeated speciation, thereby creating an ecosystem with a complex structure^36,37^. In this study, our model reproduced the process (see Fig. 2 for the simulation procedure; for details, see “Method” and Appendix 2). In this model, the evolution of the island starts from one grass species and one seabird species (for simplicity, it is assumed that the soil has already been formed on which plants can grow). At a certain interval (every 100 time-steps), one species is selected from the system and allowed to speciate. The characteristics of the offspring species are determined by giving a certain degree of mutation to the ancestor species (see Appendix 2). Also, on rare occasions (once in 1000 time-steps), brand-new species (grass, trees, seabirds, herbivorous invertebrates, carnivorous invertebrates, litter feeders, and scavengers) are immigrated from outside of the model island. It was assumed that large vertebrates were not introduced because the ecosystems of oceanic islands often lack them (disharmony^36^) (no large vertebrates other than feral goats existed on Nakoudojima Island). This was repeated for 100,000 time-steps to create an ecosystem with the primitive state (before the invasion of alien species) (Figs. 1 and 2). After that (at the 100,000th time-step), the evolution of ecosystem was stopped, and the ecosystem was left as it is during 100,000 time-steps to settle the model ecosystem to an equilibrium state. This simulation was repeated 5000 times.

**Figure 3:**
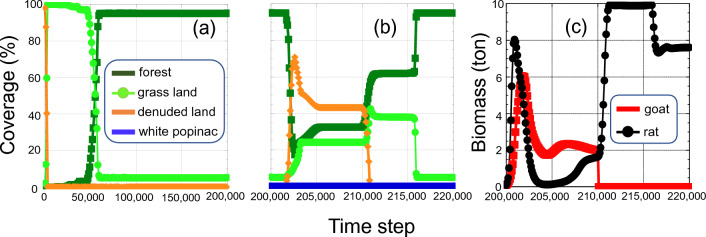
An example of changes in a model island ecosystem. (**a**) Shows the vegetation change from start to 200,000th step (just before invasions of alien species). (**b**) Shows it during the era of alien species (from 200,000th step to 220,000th step). (**c**) Shows the changes in biomass of goats and rats. In (**a**), data are plotted per 1000 time-steps and, in (**b**) and (**c**), data are plotted per 100 time-steps (this high-resolution plotting is due to high data fluctuation). In this example, white popinac did not expand. Following the invasion of white popinac, its area remained at less than 0.5%. The biomass of goats and rats rapidly increased after the invasion of these species. However, the biomass of both species soon declined because vegetation of the island declined. After the eradication of goats at the 210,000th time-step, the biomass of rats rapidly increased because rats were freed from competition with goats. The island became fully covered with forests at 216,000 time-steps. The vegetation ratio after that until the end of the simulation (310,000 time-steps) did not change. The plots after 220,000 time-steps, therefore, are omitted.

Figure 3 shows a typical result of simulation. Grassland spread very rapidly at the start of the simulation (Fig. 3a). Then, arboreous plants were immigrated and gradually increased their area, the forest area rapidly increased at around the 50,000th time-step, and the entire island was covered with forests in most cases (85.2%, 4259/5000) immediately before the invasion of the alien species (at the 200,000th time-step) (Fig. 2: Check point 1; Fig. A.2). (In this study, the upper limit of forest cover was set at 95% of the total area of the island because the island has places where trees cannot grow, such as coastal strips, along cliffs, and rocky strips. The island was considered fully covered with forests when the forest area reached this upper limit.) The only information available on the real island’s ecosystem at that time is that the entire island was covered with forest^10–13^, and then more detailed comparison of simulation results and characteristics of the real ecosystem of the island was not possible. Although we cannot say we successfully reproduced the primitive ecosystem just because the model reproduced the island fully covered with forest, this model possesses potential for reproducing the primitive ecosystem and has successfully put itself at the starting line for further development.

Alien species, i.e., goats, rats, white popinac, and Korean lawn grass, were introduced to the 4259 ecosystems in which the entire island was covered with forest, and the ecosystems were left to stand for 10,000 time-steps (Fig. 2). On actual Nakoudojima Island, following the invasion of alien species, forests did not disappear but were greatly reduced in size, and grassland and denuded ground increased^11–13,15,16^; in the simulation, similar patterns were confirmed 1303 times (Figs. 3b and A.2). Focusing on these cases, we compared the simulation results with the observed values for Nakoudojima Island: vegetation ratio^17^, number of seabirds^13,18^, and biomass of goats^38^ (Fig. 2: Check point 2). The average forest area of the model was very close to the actual value (Table A.1, Fig. A.3). The model generated a close value for grassland area although the simulated average grassland area was slightly larger than the observed area. The number of seabirds in the model ecosystem nearly matched the observed value (Table A.1). However, the denuded ground area in the model was around 40% of the observed area, and the biomass of goats was also around 40% of the observed value (Table A.1). Therefore, this evolution model did not perfectly reproduce all observed values for the ecosystem of Nakoudojima Island just before the eradication of feral goats. Because the ecosystem in the evolution model evolves freely after the initial settings, it is extremely difficult to precisely guide the model ecosystem to the target (the ecosystem at the time cross-section that needs to be reproduced). In other words, models that do not contain evolution have the target close to the start point, whereas evolution models have the target in the far-distant future. It is, therefore, difficult for an evolution model to reproduce the targeted state of the ecosystem. Nevertheless, it is noteworthy that the model could reproduce a state very close to the actual state.

Using the 1,303 ecosystems, we ran a simulation in which goats were eradicated from the model ecosystem, as had been done on Nakoudojima Island, and monitored changes in the ecosystem after the eradication. The vegetation ratio when sufficient time passed after goat eradication (after 100,000 time-steps—10 times the period when the alien species were disturbing the ecosystem; Fig. 2) showed that only in a small number of cases (197/1303 times) were forests restored to the extent that they covered the entire island and that the forest cover ended up below 50% in many cases (768/1303 times) (Figs. A.2 and A.4).

**Figure 4:**
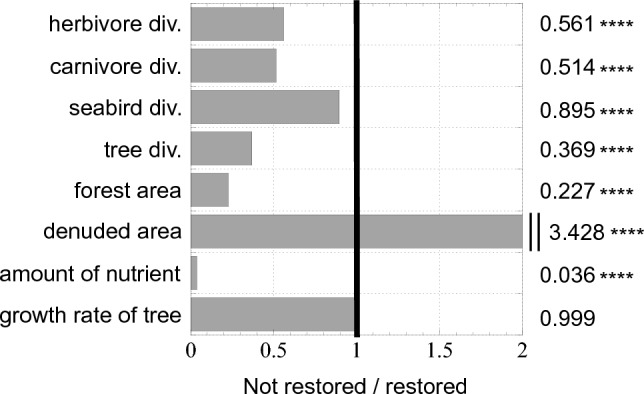
Comparison between ecosystems restored and not restored the forested state after the eradication of feral goats. “1” indicated by the bold line in each graph means there is no difference between ecosystems restored and not restored the forested state. Levels on the right side of the line indicate that the value of ecosystems not restored is greater than those restored. The values at the right end of the graph were obtained by dividing the values for the unrestored ecosystems by the values for the restored ecosystems. * indicates significance level (****p < 0.0001; ***p < 0.001; **p < 0.01; *p < 0.05). For actual values, see Table A.2.

What are the differences between the ecosystems in which forests were restored after the eradication of feral goats and those in which forests were not? When the state before the eradication of feral goats was compared between the two types of ecosystems, the ecosystems with forests not restored had much smaller numbers of animal and plant species and a much smaller amount of biomass (Fig. 4, Tables A.2 and A.3). It should also be noted that the unrestored ecosystems had a much lower underground nutrient content than the restored ecosystems (Fig. 4, Tables A.2 and A.3). However, among the cases, there were no differences in the growth rate of arboreous plants that composed the ecosystem (Fig. 4, Tables A.2 and A.3). In short, the determining factor as to whether forests were restored was not the difference in the characteristics of arboreous plant species but low underground nutrient content and low numbers of animal and plant species. Earlier studies suggested that one cause of delayed restoration of forests was oligotrophic soil^19,20,22,39^. This was also true in the model. However, the oligotrophic state of the model ecosystem was not the only cause of the reason for forests not being restored. Because the biomass of seabirds was restored following the eradication of goats (Tables A.2 and A.3), nutrient content was sufficiently high in the final state of the ecosystem after the passing of sufficient time, even for ecosystems in which forests were not restored (Tables A.2 and A.3, final state).

To determine why forest was not restored after the restoration of nutrient content following the eradication of feral goats, we analyzed changes in the ecosystem after the goat eradication and identified four patterns for the ecosystems in which forests were not restored, depending on the type of damage caused by alien species (Fig. A.5). The first, which was rare (18/768 times), was a pattern in which all arboreous plants became extinct after goat eradication. Characteristically, these ecosystems had an especially low nutrient content, a larger forest area compared with the other ecosystem patterns (and most forests were pure white popinac), and a higher number and total biomass of herbivorous animal species (Table A.5). Pure white popinac forests are formed in an extremely oligotrophic environment because white popinac has nitrogen-fixing activity^34^ (see the last paragraph in section 1-1 in Appendix 1), allowing them to grow to some extent in an extremely oligotrophic environment where other arboreous trees cannot survive. If feral goats are eradicated from this state, white popinac, now free of the feeding pressure from feral goats, rapidly increases its cover (Fig. A.6a); however, the biomass of herbivores, which feed on white popinac, also rapidly increases (Fig. A.6b), thereby quickly reducing the size of white popinac forest. This phenomenon is often observed in the predator–prey relationship^40,41^. It is considered that white popinac forest disappeared as a result. Alien species exterminate many constituent species of an ecosystem and disrupt the ecosystem balance, and the worst result is this pattern.

The second (212 out of the 768 ecosystems in which forests were not restored) is the pattern in which white popinac is extremely dominant in forests (white popinac forests account for over 90% of the total forest area) formed following goat eradication, and such forests reach equilibrium without covering the entire island (Fig. A.7). Most of these cases occurred when the diversity of the eventually surviving arboreous plants was low (three species or less). Also, in 177 out of the 212 ecosystems, white popinac was the only surviving arboreous plant species. In addition, the ecosystems with this pattern were characterized by especially low numbers of surviving herbivorous and carnivorous species (Table A.5). Therefore, we suggest that the ecosystems consisting of a small number of species including white popinac reached equilibrium with their biomasses insufficiently large to cover the entire island. (It is known that ecosystems with low diversity are prone to reach equilibruium^42–45^.)

The third (289 out of the 768 ecosystems in which forests were not restored) is the pattern in which a small number of arboreous plant species, not including white popinac, reach equilibrium without covering the entire island. The characteristics of these ecosystems were similar to those of the second pattern. Specifically, the diversity of eventually surviving arboreous plant species was three species or less, and the diversities of herbivorous and carnivorous animal species were low (Table A.5). This suggests that the ecosystems consisting of a small number of species reached equilibrium, as did the ecosystems of the second pattern. Because the forests were composed of tree species with a growth rate lower than that of white popinac, the final forest area tended to be smaller than in the second pattern (Table A.5).

The last (289 out of the 768 ecosystems in which forests were not restored) is the pattern in which biomass of arboreous plants was kept at a low level despite the relatively high diversity of surviving arboreous plants. In these ecosystems, more than three arboreous plant species survived. The eventual biomass per arboreous plant species in these ecosystems remained at approximately one-sixth of that of the restored ecosystems (Fig. A.8, Table A.6). Now, the question arises, what hindered the growth of arboreous plants? The answer is the feeding damage caused by herbivores. The final number of herbivore species was higher in these ecosystems than in restored ecosystems, remaining at two-thirds of the number of carnivores (Fig. A.8, Table A.6). This result suggests that in the not restored ecosystems, herbivores were relatively free of predation pressure from carnivores. In fact, the biomass of herbivores per arboreous plant species in these ecosystems was 1.5 times that in the restored ecosystems (Fig. A.8, Table A.6), indicating that arboreous plants were severely damaged by herbivores. This is the reason that forests were not restored. As described above, oligotrophic ecosystems do not have sufficient primary production and thus are especially vulnerable to disturbance by alien species (Tables A.2 and A.3). Alien species exterminate many species that constitute an ecosystem and destroy its resilience. This is why its forest is not restored even if alien species are eradicated.

Why and when did the ecosystems that were eventually not restored become deficient in nutrient? When the state before the invasion of alien species was confirmed by going backward in time, the ecosystems that were not restored already had an amount of nutrient that was an order of magnitude lower than for ecosystems where forests were restored (Fig. A.9, Tables A.2 and A.3: before invasion) although the nutrient concentration was not low enough to affect plant growth. Comparison of the characteristics of arboreous plants at this point showed that the ecosystems that were not restored consisted of arboreous plant species with a high growth rate (Tables A.2 and A.3: before invasion). We suggest that because arboreous plant species with a high growth rate require large amounts of nutrient, in ecosystems dominated by such species, nutrient moved to the plants, causing nutrient deficiency in the soil. This process is believed to be a cause of oligotrophic soil in tropical rainforests^37,46^, with the soil becoming oligotrophic because plants grow at a high rate in this environment and most of nutrient are kept by the aboveground part of plants. Such a process tropical occurring in the subtropical Ogasawara Islands is quite possible. Also, these arboreous plant species with a high growth rate selectively became extinct following the invasion of alien species (Fig. A.10, Tables A.2 and A.3: basic growth rate for tree).

When does the growth rate start varying among the constituent species? The answer is “at the beginning.” Comparing the characteristics of the constituent species, by going backward in time, showed that whether forests were restored after eradication of goats depended on the characteristics of arboreous plant species colonizing in the early stage of ecosystem evolution (Fig. A.11). In the ecosystems in which arboreous plant species with a high growth rate colonize first, large amounts of nutrient are consumed by them, and underground nutrient become exhausted, making the soil oligotrophic. As a result, many species become extinct as they cannot withstand the disturbance caused by alien species, the ecosystem loses its resilience, and the forest is not restored after eradication of the alien species. In short, the founder effect determines the distant future after the eradication of alien species.

Why does the growth rate of first-colonizing arboreous plant species not change significantly during the evolution process and keep affecting the outcome until the end? In fact, our analysis showed no statistically significant differences between the growth rate of arboreous plant species colonizing during the early state (before 10,000 time-steps) and the growth rate of arboreous plant species after evolution (immediately before the invasion of alien species) (Fig. A.12, Table A.7). One possible reason is that immigration of new species is a rare event. The first-colonizing arboreous plant species occupies the island before the next arboreous plant species arrives. It is therefore difficult for subsequently immigrated arboreous plants to increase and replace the first-colonizing species. Another reason is that changes in growth rate work both to the advantage and disadvantage of the survival of arboreous plant species. For example, an advantage of species with a high growth rate is that they can grow rapidly and thus gain the upper hand in competition with other plant species; however, a disadvantage is that they are susceptible to high feeding pressure from herbivores (Fig. A.13). Because species with a high growth rate provide a large amount of food to herbivores, herbivores tend to evolve to feed on such species. We suggest that the growth rate of arboreous plants does not change significantly during their evolution process for these reasons.

What will the future of Nakoudojima Island be like? The results of this study predict that it will take a very long time before the entire island is covered with forest following goat eradication. Even the restored ecosystems typically go through a stage in which they increase forest area very slowly after goat eradication as if they are about to reach equilibrium (e.g., Fig. 3b). The causes are that, immediately after goat eradication, ecosystems are oligotrophic and only arboreous plants with a low growth rate are left (Fig.A.10, Tables A.2 and A.3: basic growth rate for tree). Especially in cases where forest reduction rate is high, restoration tends to take time (Fig. A.14). The examination of 111 cases in which forest area was reduced to less than 20% (as was the case with Nakoudojima Island) and then recovered to the point of the entire island being covered with forest following goat eradication showed that it took 33,479.28 time-steps (s.d.: 20,116.7) on average. This is more than three times the period when feral goats existed on the island (10,000 time-steps). Forests were restored within 10,000 time-steps only 14 times. For Nakoudojima Island, the fact that its forests have not been restored more than 20 years after the eradication of goats is considered problematic^20–22^; however, this may be unavoidable at this point.

Will the forest of Nakoudojima Island be restored in the future? The likelihood of its restoration becomes higher if the distant future is included, but the probability of the entire island becoming covered with forest is still around 15% (197/1303 times; Figs. A.2 and A.4). In addition, because the soil of Nakoudojima Island is oligotrophic^19,20,22,39^ and white popinac forests are gradually expanding there^21^, it is predicted that forests will not recover to the extent that they cover the entire island and that it will be partially covered with forests dominated by white popinac (i.e., the aforementioned second pattern).

Our results show that forests can recover to the extent that they cover the entire island even if the growth rate of arboreous plants is low, if nutrient are sufficiently supplied, the diversity within the ecosystem is high, and the balance among plants, herbivorous animals, and carnivorous animals is maintained (Fig. 4, Tables A.2, A.3, and A.6). However, we should not simply be overjoyed even if forests have been restored. In this study, out of the 197 simulations in which forests were restored, 58 contained zero herbivore species at the final state (Table A.8). In these cases, forests were restored only because of the lack of feeding pressure from herbivores, and the ecosystem by no means was restored to its original state. Also, when targeting the cases in which forests were restored after eradication of feral goats, the comparison between ecosystems in their pre-eradication and final states showed that the number of herbaceous plant species and carnivore species significantly decreased (Fig. A.15). The decrease in the number of herbaceous plant species may be attributed mainly to post-eradication vegetation changes from grassland to forest, as grassland area is greatly reduced as a result of forest expansion. It should also be noted that the biomass of rats increased around 8.65 times after goat eradication (Fig. 3c, Tables A.2 and A.3). This was caused by an increase in the biomass of animals and plants following goat eradication, thereby greatly increasing food for rats—generalist predators (Tables A.2 and A.3). Rats are widely known to strongly disrupt the ecosystems of oceanic islands^47–51^, and it is believed that the species diversity was markedly reduced by the direct predation by rats and by competition with rats over food. As pointed out in earlier studies that used models that did not contain evolution^3,7,8^, this result indicates that it is effective from the viewpoint of ecosystem conservation to simultaneously eradicate multiple alien species.

Although there are 1133 cases where the island maintained its forest cover even after the invasion of alien species (Fig. A.2), the ecosystem, in these cases, did not retain its original state. During the period when alien species were disrupting the ecosystem, the species diversity and biomass of both animals and plants significantly decreased (Fig. A.16, Table A.9). Even if an island remains entirely covered with forest following invasion of alien species, we should not be tricked by its apparent state.

The ecosystem evolution model developed in this study predicted that forests on Nakoudojima Island may not be restored after the eradication of feral goats, especially within a short period. This is close to the present state of this island. The ecosystem evolution model suggested the three causes for this prediction; (1) the oligotrophic state of the model ecosystem derived from the founder effect in the distant past (Fig. A.11), (2) arboreous plant species with a high growth rate selectively became extinct (Fig. A.10; in addition to the oligotrophic state, herbivores tend to evolve to feed on such species), (3) the ecosystem lost its resilience because many species became extinct due to the alien species. In contrast, the previously published model without evolutionary processes^8^ predicted that forests on the island will be quickly restored after eradication of feral goats—this prediction does not match the current situation on the island. This mismatch is because the model without evolutionary processes cannot reproduce the former two causes mentioned above, which occurred in the middle of ecosystem evolution. In addition, the third cause was also reinforced by the first cause (the oligotrophic state, Fig. 4, Tables A.2 and A.3).

This study showed a possibility that an event in the far-distant past could determine the future of an ecosystem. As discussed, the biggest advantage of our ecosystem evolution model is that we can analyze causal relations while freely moving within the time base. Also, there is a possibility that various events determine the fate of an ecosystem, evolving the ecosystem to various states, and our ecosystem evolution model enables the exploration of many such “parallel worlds.” Although this study focused on a case similar to that of Nakoudojima Island, our model is applicable to the “world” in which an island remains entirely covered with forests following invasion of alien species as well as the “world” in which an island completely loses its forests. This characteristic of our ecosystem evolution model will help us understand current ecosystems under various conditions as well as help us develop ecosystem conservation policies based on ideas that cannot otherwise be obtained from static models that do not incorporate evolution. We are not denying the application of models that do not contain evolution, and many valuable findings have been gained using such models^9^. Models that contain evolution have their own disadvantages, such as the difficulty in reproducing the ecosystem target state and requiring calculation time that is an order of magnitude longer. Therefore, there will continue to be cases where models that do not contain evolution are necessary. However, if phenomena cannot be explained using such models, why not try an ecosystem evolution model?

## Method

### Outline of the ecosystem model

Our model reproduces the nutrient (e.g. nitrogen) cycle in the ecosystem of Nakoudojima Island in the Ogasawara (Bonin) Islands. This cycling is described schematically in App.1-Fig. 1 in Appendix 1. This island ecosystem consists of seabirds, plants (trees and grasses), and invertebrate animals [primarily arthropods, we assume that the feeding types are herbivorous, carnivorous, scavenger, and litter-eating]. Each of these feeding groups contains multiple species. In addition, three invasive species are considered: feral goats, rats, white popinac (*Leucaena leucocephala*). Explicit spatial structures are not considered in this model. This is because Nakoudojima Island is small with an area of approximately 1.38 km^2^ and is relatively flat with few undulations.

It is assumed that plants need multiple types of nutrients and that required nutrient types change depending on the maturation stage of the ecosystem. However, since the model becomes very complex if the dynamics of multiple types of nutrients are considered, nutrient are treated collectively, not individually. To express nutrient contents within organisms, nitrogen values^52–54^ are used for convenience.

Including parameters for species (e.g. growth rate, death rate, nutrient content, and feeding type), this model requires many variables to be determined (App.1-Tables 1 and 2 in Appendix 1). Values for the variables were obtained from the literature, unpublished data, experts’ opinions, and computational constraints (App.1-Table 1 in Appendix 1). For other variables (App.1-Table 2 in Appendix 1), we chose the parameter sets that best reproduced the vegetation ratio^17^ and the biomass of goats^38^ of the ecosystem of Nakoudojima Island from 500 parameter sets, of which values were determined randomly, via 200–1000 iterations of simulation (for details, see Appendix 2 in Yoshida et al.^55^). For plant species, ecological traits of individual species are set by applying the concept of Grime’s triangle^26–28^ (for details, see the Main text and section 1-1 in Appendix 1).

Our model incorporates the following three processes: growth of animals and plants regulated by nutrients, inter-specific interactions (predator–prey, competition, and interference), and detritus decomposition (App.1-Fig. 1 in Appendix 1).

The nutrient cycle on oceanic islands is characteristically a closed system in a small area. On the Ogasawara Islands, the input of nutrient from seabirds is a major nutrient source^39^. Seabirds feed on fish at sea and leave droppings and die on islands. Droppings and carcasses of seabirds decompose and are broken down into nutrients available to plants and are stored in belowground sinks (see section 8 in Appendix 1).

Plants grow by absorbing nutrients from under the ground (see section 1-2 in Appendix 1). Plants absorb as much nutrient as they need at the time. The amount of biomass growth is regulated by the amount of nutrients absorbed. In a severely oligotrophic condition, plant biomass may be reduced.

A similar situation applies to animals. They assimilate as much nutrient as they need at the time (see section 3 in Appendix 1). Animals, except for detritivores, assimilate nutrients via predator–prey interactions, which are set based on the Niche Model^29^; that is, predators feed on other species with characteristics that fit their preference (see section 3-1 in Appendix 1). Changes in biomass regarding predator–prey interactions are described by the following Holling type III equation^30^:

$$\frac{\mathrm{d}{M}_{1}}{\mathrm{dt}}=\frac{k{M}_{1}{{M}_{2}}^{2}}{h+{{M}_{2}}^{2}},$$

where *M*_*1*_ and *M*_*2*_ are the amount of biomass of species 1 (predator) and 2 (prey), respectively; *k* is an interaction coefficient when species 1 (predator) feeds on species 2 (prey); and *h* is a constant representing handling time^56^. A predator selectively allocates their feeding effort to prey with characteristics close to the predator’s feeding preference. A predator sequentially feeds on prey according to the difference between the feeding preference of the predator and the characteristics of prey (App.1-Fig. 2 in Appendix 1). A predator stops feeding when its nutrient intake reaches the amount that they need at the time, even if there is prey remaining. Animals assimilate a part of the biomass taken in. The assimilation rates of animals were determined by referring to the literature (see section 3-1 in Appendix 1).

The biomass not assimilated by a predator is expelled as droppings. In addition, animal carcasses and litter are produced constantly. A part of detritus [carcasses and litter] is used by detritivores (scavengers and litter feeders; see section 4 in Appendix 1). Unused detritus is decomposed within a certain period and broken down into nutrients available to plants and stored in belowground sinks (see section 8 in Appendix 1).

Our model incorporates competition in several forms: competition among plant species and seabird species, disturbance of seabird nests by goats, and trampling of plants by goats and seabirds (App.1-Fig. A.1 in Appendix 1). Plants having similar characteristics compete against each other (see section 1-4 in Appendix 1). Inter-specific competitions are set based on the Niche Model^29^ as for predator–prey interactions between animals mentioned above. Inter-specific competition between plant species is described by a process in which a taller plant (species 1) encroaches the habitat area of a smaller plant (species 2). The size of the area encroached is calculated by the following simple Lotka–Volterra equation (see section 1-4 in Appendix 1):

$${dA}_{2}={E}_{1}\times {A}_{1}\times {A}_{2}\times {H}_{12}\times {C}_{s2},$$

where *dA*_*2*_ is the size of area of species 2 encroached by species 1; *A*_*1*_ and *A*_*2*_ are area occupied by species 1 and 2, respectively; *E*_*1*_ is the area increase rate of species 1; *H*_*12*_ is a height difference coefficient; and *C*_*S2*_ is the index of stress tolerance of species 2. The value of *dA*_*2*_ is larger as the difference between the height of the two plant species is greater and as the stress tolerance of the smaller plant species is lower. Our model assumes that herbaceous plants are shorter and so cannot compete with trees. Competition among seabird species is described as the same process (see section 7 in Appendix 1). In this case, a bigger seabird encroaches the habitat area of a smaller seabird. Seabirds interfere with plant growth around their nests by trampling (see section 7 in Appendix 1). Goats interfere with the nesting of all seabird species by trampling a wide area on an island (see section 6 in Appendix 1).

One of the characteristics of island ecosystems is that they are closed systems and limited in area (see the fifth paragraph in section 1-1 in Appendix 1). This means that the total amount of plants that an island can support is limited, which in turn limits the total amount of animals that can be supported. These are essential considerations when modeling the nutrient cycle of an island. Our model assumes that the habitat area of plants and the nesting area of seabirds are finite, whereas our model does not use an explicitly specific structure but incorporates the concept of area. Then, competition among plant species and among seabird species is described in terms of competing for space.

### Explanation of the ecosystem evolution

An oceanic island that has suddenly formed far from other land does not initially have living things; however, through repeated rare immigrations of living things and their speciation on the island, the island will gradually increase its biodiversity, and the ecosystem on the island evolve to have a complex structure^36,37^. This process is reproduced in our model (for details, see Appendix 2).

In this study, the simulation starts with one species of seabird and one species of herbaceous plant. For simplicity, it is assumed that the soil on which plants can grow has already been formed. The ecosystem is made to evolve during 100,000 time-steps. At every 1000 time-steps in the course of evolution of a model ecosystem, a completely new species immigrates to the ecosystem. Immigrations of a tree, grass, herbivore, carnivore, scavenger, litter feeder, and seabird occur randomly. Variables and interactions of the new species are given in the same manner as in the initial setting (see Appendix 1), except for the initial biomass. The initial biomass of immigrant is set to one tenth of the case of initial setting, because the number of individuals of immigrant to an oceanic island may be very small.

Once in 100 time-steps, one species is selected from the constituent species of the ecosystem, and this species is given the opportunity for speciation. The opportunity for speciation is given to the categories in the following order: herbaceous plants, arboreous plants, herbivorous animals, and carnivorous animals. Which species speciate within each category is randomly determined. The total biomass of the new species is set to 5% of that of its ancestor species. Scavengers, litter feeders, and seabirds are treated as environmental elements, and although they immigrate, they are not given the opportunity for speciation. The reason is that these types of species never become extinct due to lack of food as they feed on detritus (detrivores) or fish at sea (seabirds) and their species numbers increase unlimitedly when given the opportunity for speciation.

Variables of the new species are set by adding small values (slight mutations) to those of its ancestor. Values are given for the variables by random numbers drawn from Gaussian distributions with a mean of 0. The standard deviations of the Gaussian distributions are based on the evolutionary rate (E) of the ancestor (see Appendix 2-Table 1). These standard deviations are set to about 10% of the mean of each variable, except for C, S, and R for plant species (for C, S, and R for plant species, see section 1-1 in Appendix 1). Gradual evolution is thereby realized in the model. The variable values not shown in Appendix 2—Table 1 are automatically determined by the variable values listed in Appendix 2—Table 1 (see Appendix 1 for details). Nutrient contents of new species are the same as those of their ancestor species.

At the 100,000th time-step, the ecosystem evolution is stopped, and the ecosystem is left as it is during 100,000 time-steps to settle the model ecosystem to an equilibrium state.

Alien species – goats, rats, white popinac, and Korean lawn grass—are made to invade this resultant primitive ecosystem. During 10,000 time-steps, the ecosystem is disrupted by these introduced species. Then, the feral goats are eradicated, as had been done on Nakoudojima Island. Following the goat eradication, the ecosystem is left as it is during 100,000 time-steps, and post-goat eradication changes in the ecosystems are analyzed. For details, see Appendix 2.

### Simulation of eradication of invasive goats

The eradication began 10,000 time-steps after the invasions of alien species (210,000th time step). In simulating the eradication of goats, a constant amount of goat biomass was removed daily from the virtual ecosystem. In this research, we ran a simulation assuming that 400 kg of biomass was removed on a time-step. The simulation assumed that the eradicated goats remained in the ecosystem as carcasses as it occurred in the actual eradication project.

### Supplementary Information

Supplementary Information 1.

Supplementary Information 2.

Supplementary Figures.

Supplementary Tables.

## Data Availability

The datasets used and/or analyzed during the current study available from the corresponding author on reasonable request.
