# Supplementary material for: Predicting ecosystem changes by a new model of ecosystem evolution
Source: Sci Rep. 2023 Sep 16;13:15353. doi: 10.1038/s41598-023-42529-9 (PMC10505200; doi:10.1038/s41598-023-42529-9)
Supplement: Supplementary file 1 — Supplementary Information 1. [file 41598_2023_42529_MOESM1_ESM.zip › Appendix 1/App1_Figure 1.pptx]

## Slide 1
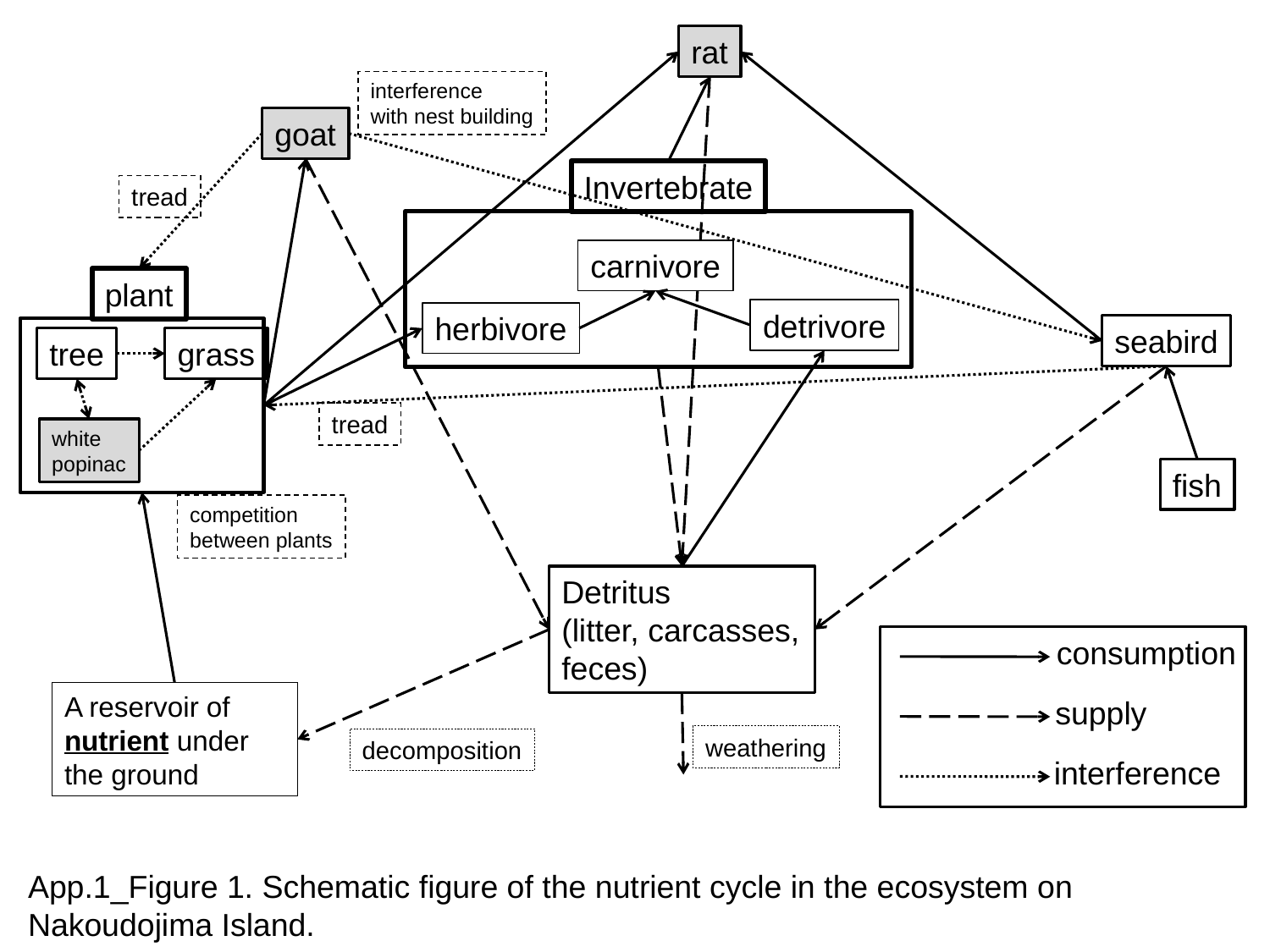

rat
interference
with nest building
goat
Invertebrate
tread
carnivore
plant
detrivore
herbivore
seabird
tree
grass
tread
white
popinac
fish
competition
between plants
Detritus
(litter, carcasses, feces)
consumption
A reservoir of nutrient under the ground
supply
weathering
decomposition
interference
App.1_Figure 1. Schematic figure of the nutrient cycle in the ecosystem on Nakoudojima Island.

## Slide 2
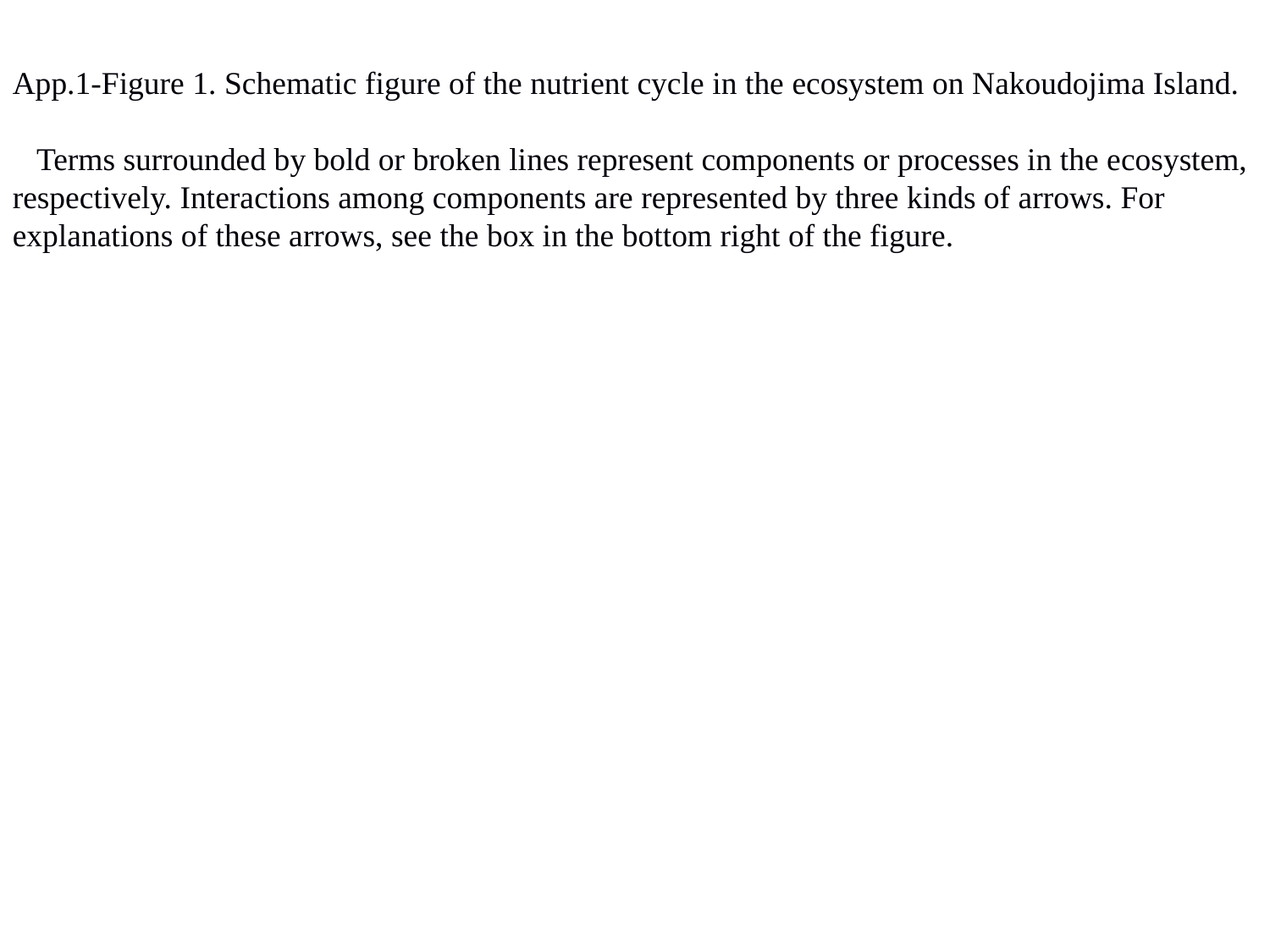

App.1-Figure 1. Schematic figure of the nutrient cycle in the ecosystem on Nakoudojima Island.
 Terms surrounded by bold or broken lines represent components or processes in the ecosystem, respectively. Interactions among components are represented by three kinds of arrows. For explanations of these arrows, see the box in the bottom right of the figure.
