# Supplementary material for: Predicting ecosystem changes by a new model of ecosystem evolution
Source: Sci Rep. 2023 Sep 16;13:15353. doi: 10.1038/s41598-023-42529-9 (PMC10505200; doi:10.1038/s41598-023-42529-9)
Supplement: Supplementary file 1 — Supplementary Information 1. [file 41598_2023_42529_MOESM1_ESM.zip › Appendix 1/App1_Figure 2.pptx]

## Slide 1
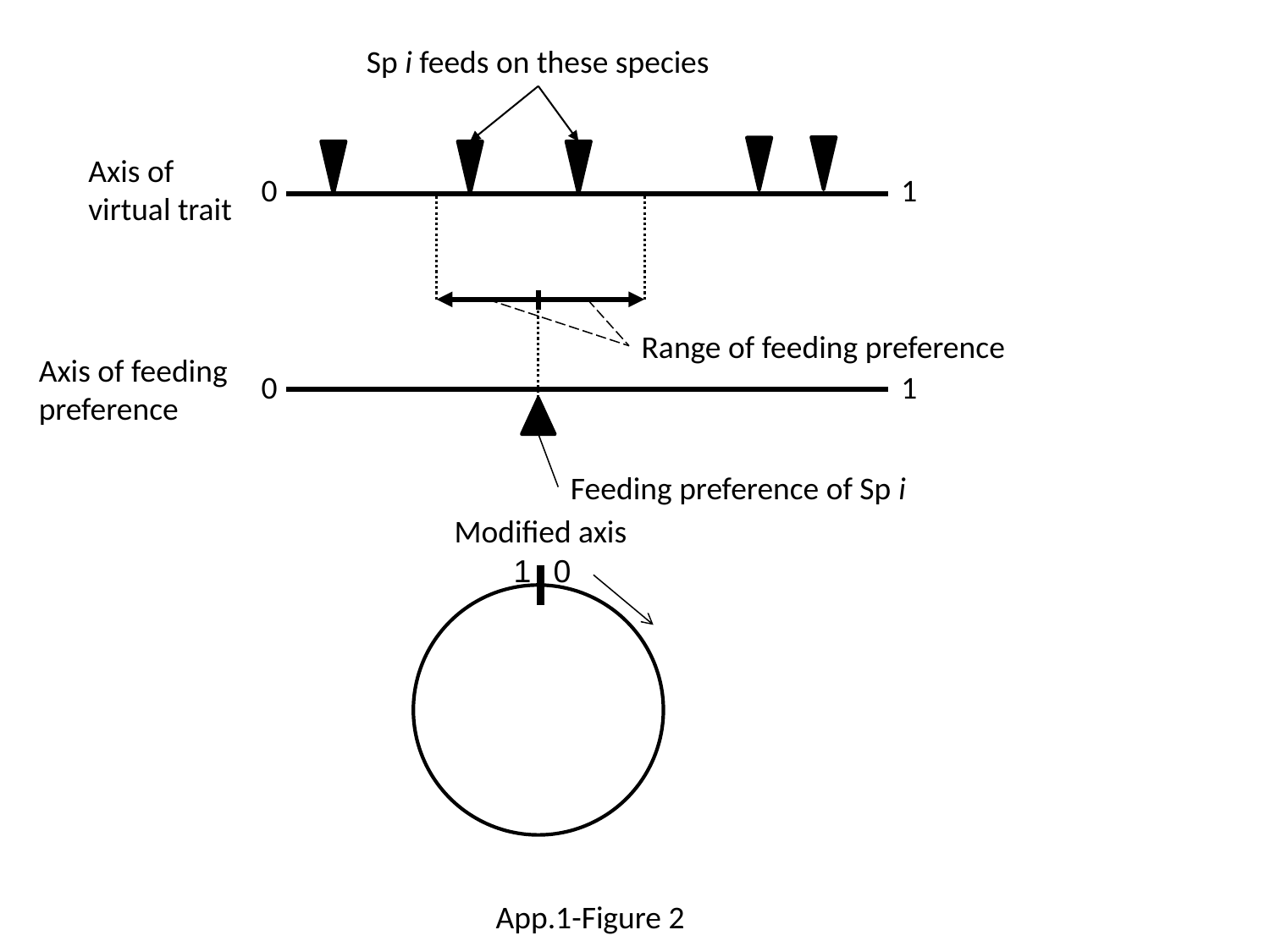

Sp i feeds on these species
Axis of
virtual trait
0
1
Range of feeding preference
Axis of feeding
preference
0
1
Feeding preference of Sp i
Modified axis
1
0
App.1-Figure 2

## Slide 2
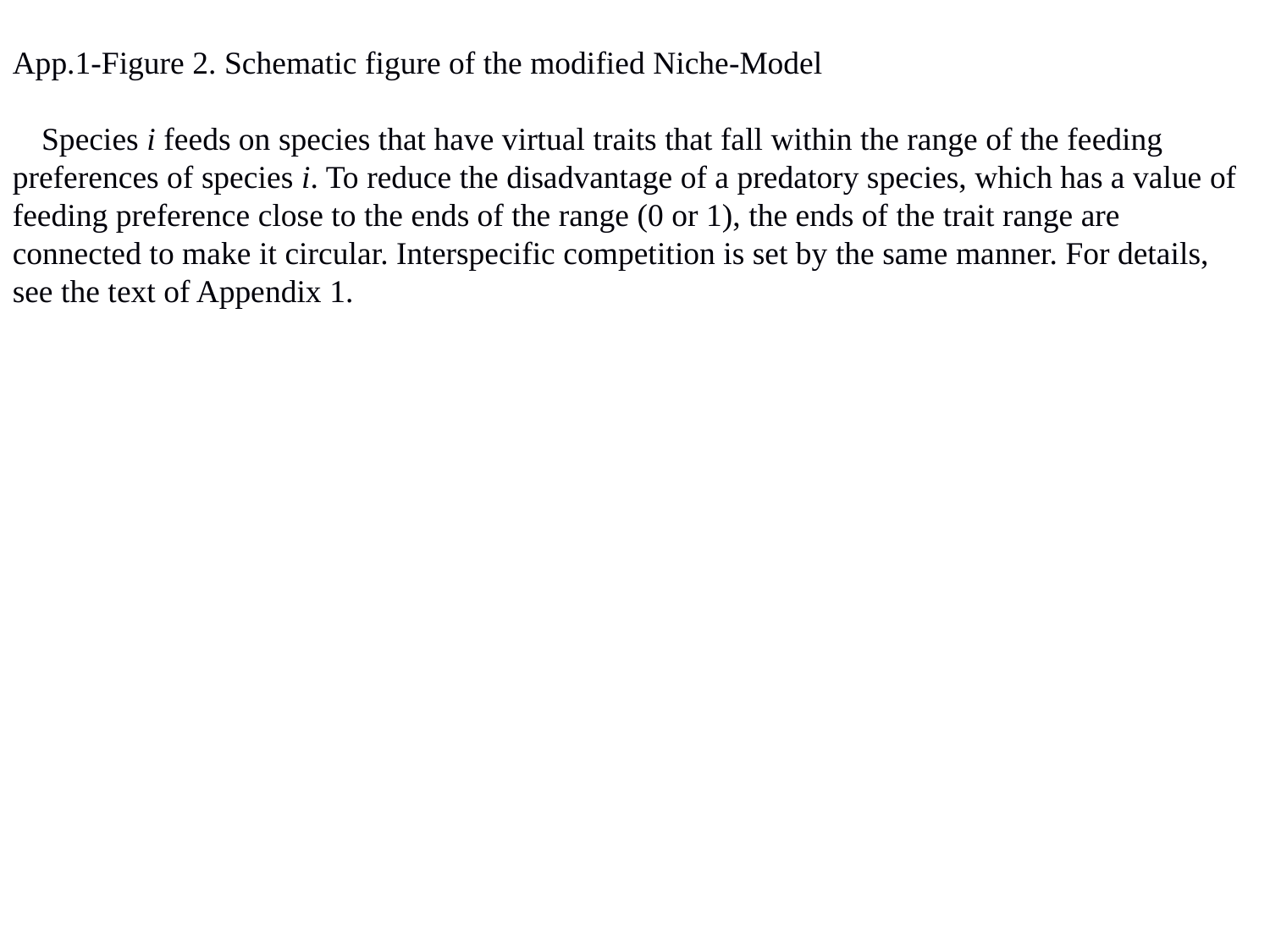

App.1-Figure 2. Schematic figure of the modified Niche-Model
 Species i feeds on species that have virtual traits that fall within the range of the feeding preferences of species i. To reduce the disadvantage of a predatory species, which has a value of feeding preference close to the ends of the range (0 or 1), the ends of the trait range are connected to make it circular. Interspecific competition is set by the same manner. For details, see the text of Appendix 1.
