# Supplementary material for: Predicting ecosystem changes by a new model of ecosystem evolution
Source: Sci Rep. 2023 Sep 16;13:15353. doi: 10.1038/s41598-023-42529-9 (PMC10505200; doi:10.1038/s41598-023-42529-9)
Supplement: Supplementary file 1 — Supplementary Information 1. [file 41598_2023_42529_MOESM1_ESM.zip › Appendix 1/App1_Figure 3.pptx]

## Slide 1
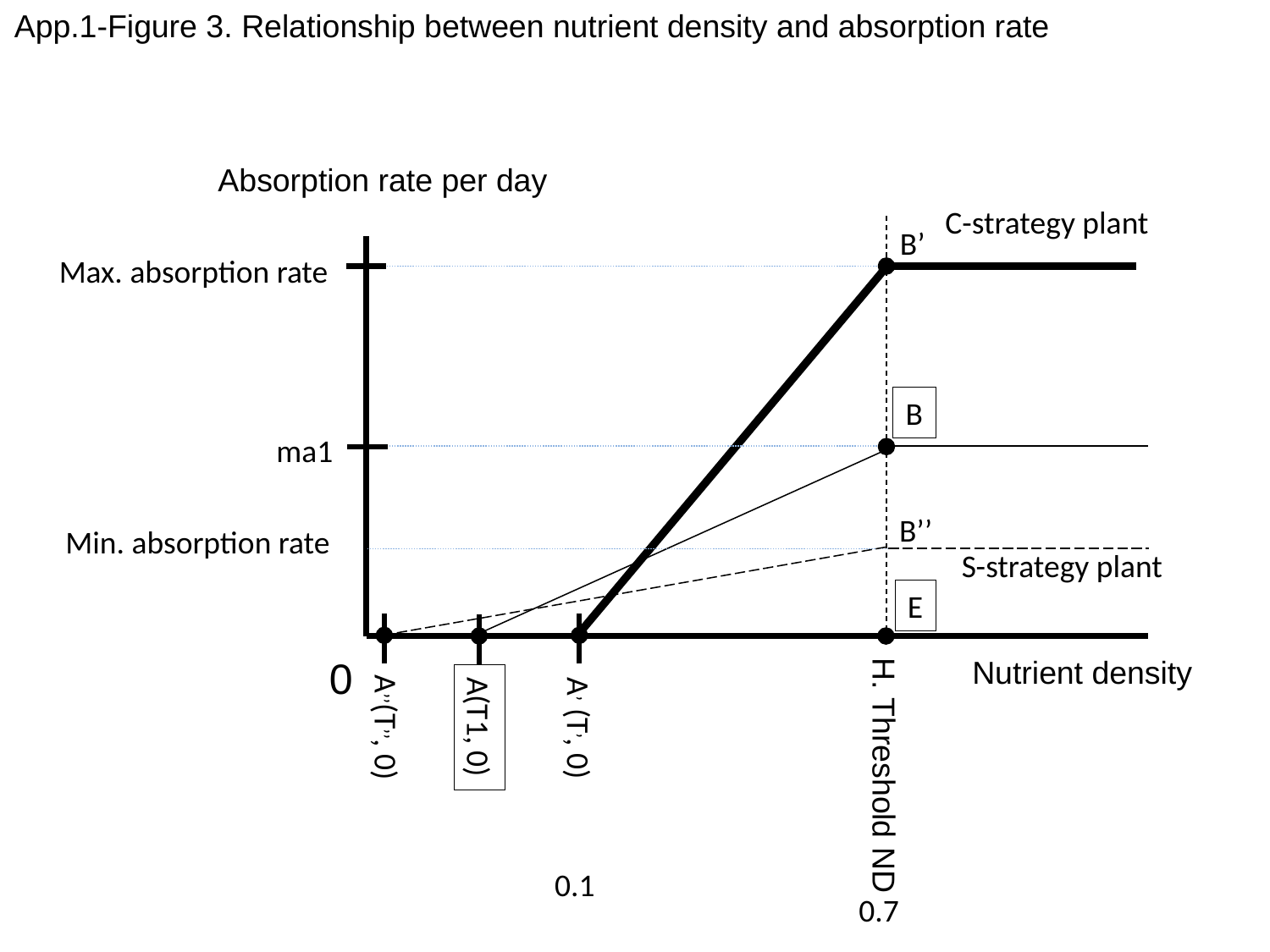

App.1-Figure 3. Relationship between nutrient density and absorption rate
Absorption rate per day
C-strategy plant
B’
Max. absorption rate
B
ma1
B’’
Min. absorption rate
S-strategy plant
E
0
Nutrient density
A(T1, 0)
A’’(T’’, 0)
A’ (T’, 0)
H. Threshold ND
0.1
0.7

## Slide 2
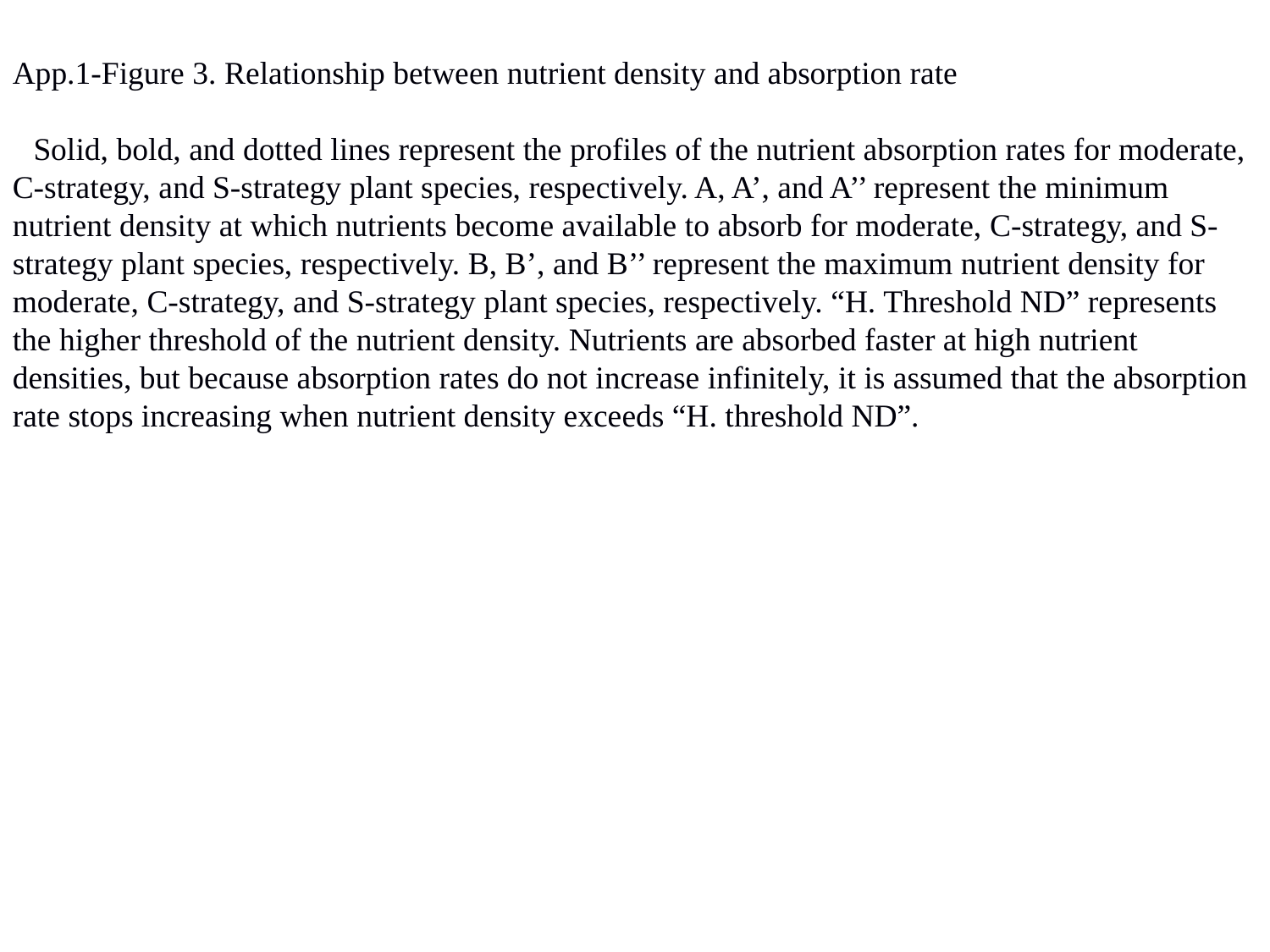

App.1-Figure 3. Relationship between nutrient density and absorption rate
Solid, bold, and dotted lines represent the profiles of the nutrient absorption rates for moderate, C-strategy, and S-strategy plant species, respectively. A, A’, and A’’ represent the minimum nutrient density at which nutrients become available to absorb for moderate, C-strategy, and S-strategy plant species, respectively. B, B’, and B’’ represent the maximum nutrient density for moderate, C-strategy, and S-strategy plant species, respectively. “H. Threshold ND” represents the higher threshold of the nutrient density. Nutrients are absorbed faster at high nutrient densities, but because absorption rates do not increase infinitely, it is assumed that the absorption rate stops increasing when nutrient density exceeds “H. threshold ND”.
