# Supplementary material for: Predicting ecosystem changes by a new model of ecosystem evolution
Source: Sci Rep. 2023 Sep 16;13:15353. doi: 10.1038/s41598-023-42529-9 (PMC10505200; doi:10.1038/s41598-023-42529-9)
Supplement: Supplementary file 1 — Supplementary Information 1. [file 41598_2023_42529_MOESM1_ESM.zip › Appendix 1/App1_Figure 4.pptx]

## Slide 1
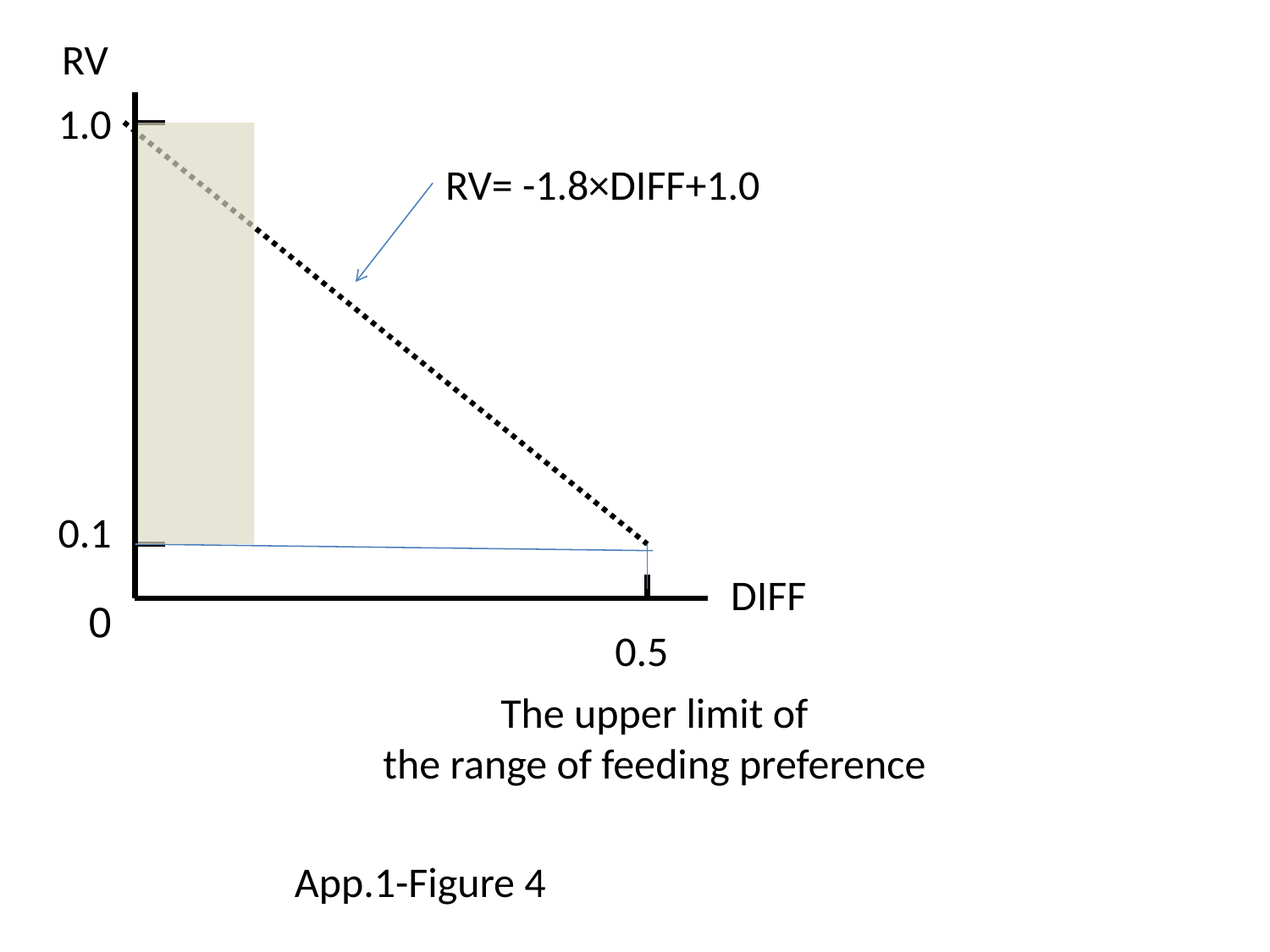

RV
1.0
RV= -1.8×DIFF+1.0
0.1
DIFF
0
0.5
The upper limit of
the range of feeding preference
App.1-Figure 4

## Slide 2
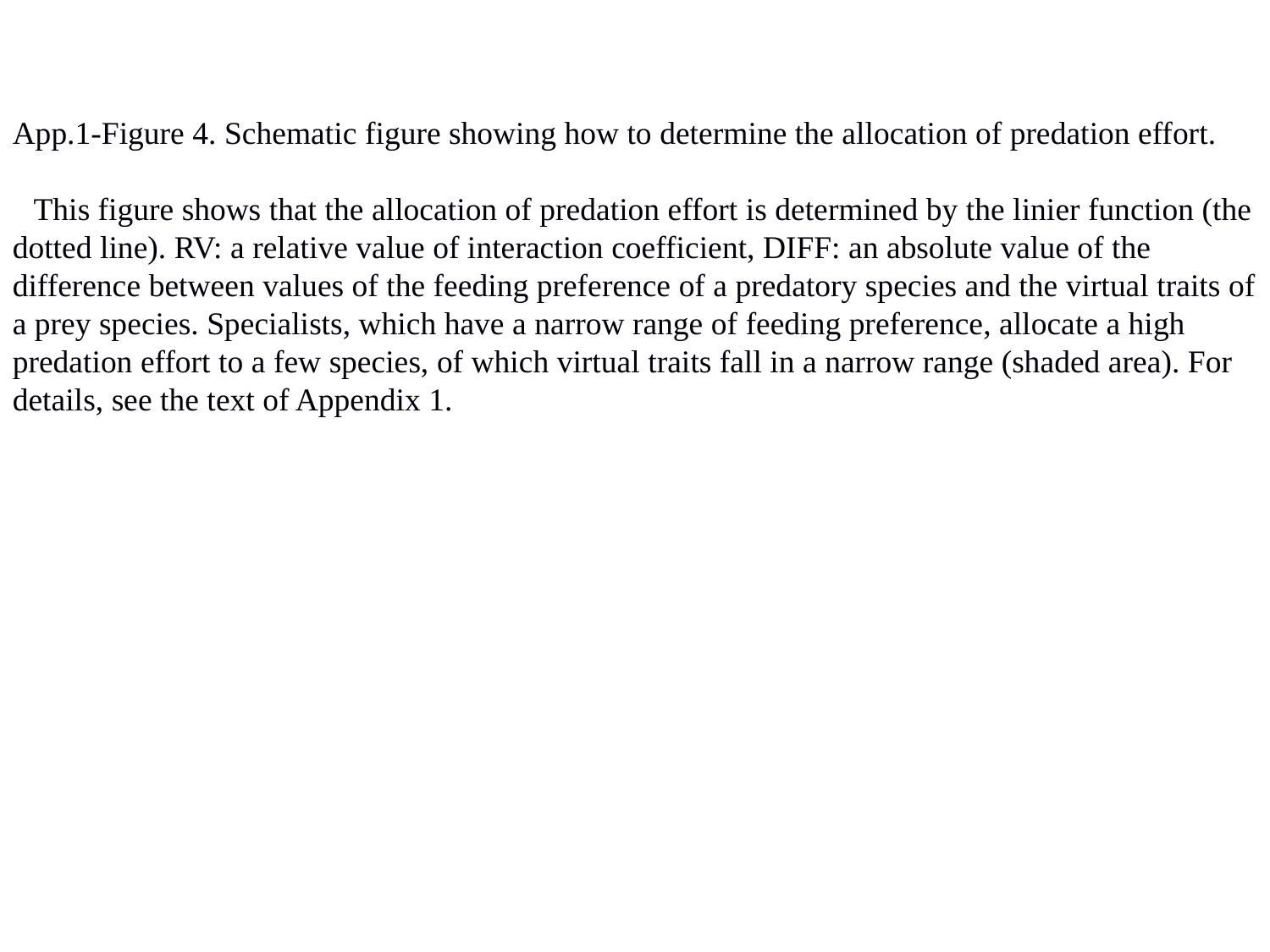

App.1-Figure 4. Schematic figure showing how to determine the allocation of predation effort.
This figure shows that the allocation of predation effort is determined by the linier function (the dotted line). RV: a relative value of interaction coefficient, DIFF: an absolute value of the difference between values of the feeding preference of a predatory species and the virtual traits of a prey species. Specialists, which have a narrow range of feeding preference, allocate a high predation effort to a few species, of which virtual traits fall in a narrow range (shaded area). For details, see the text of Appendix 1.
