# Supplementary material for: Predicting ecosystem changes by a new model of ecosystem evolution
Source: Sci Rep. 2023 Sep 16;13:15353. doi: 10.1038/s41598-023-42529-9 (PMC10505200; doi:10.1038/s41598-023-42529-9)
Supplement: Supplementary file 3 — Supplementary Figures. [file 41598_2023_42529_MOESM3_ESM.pptx]

## Slide 1
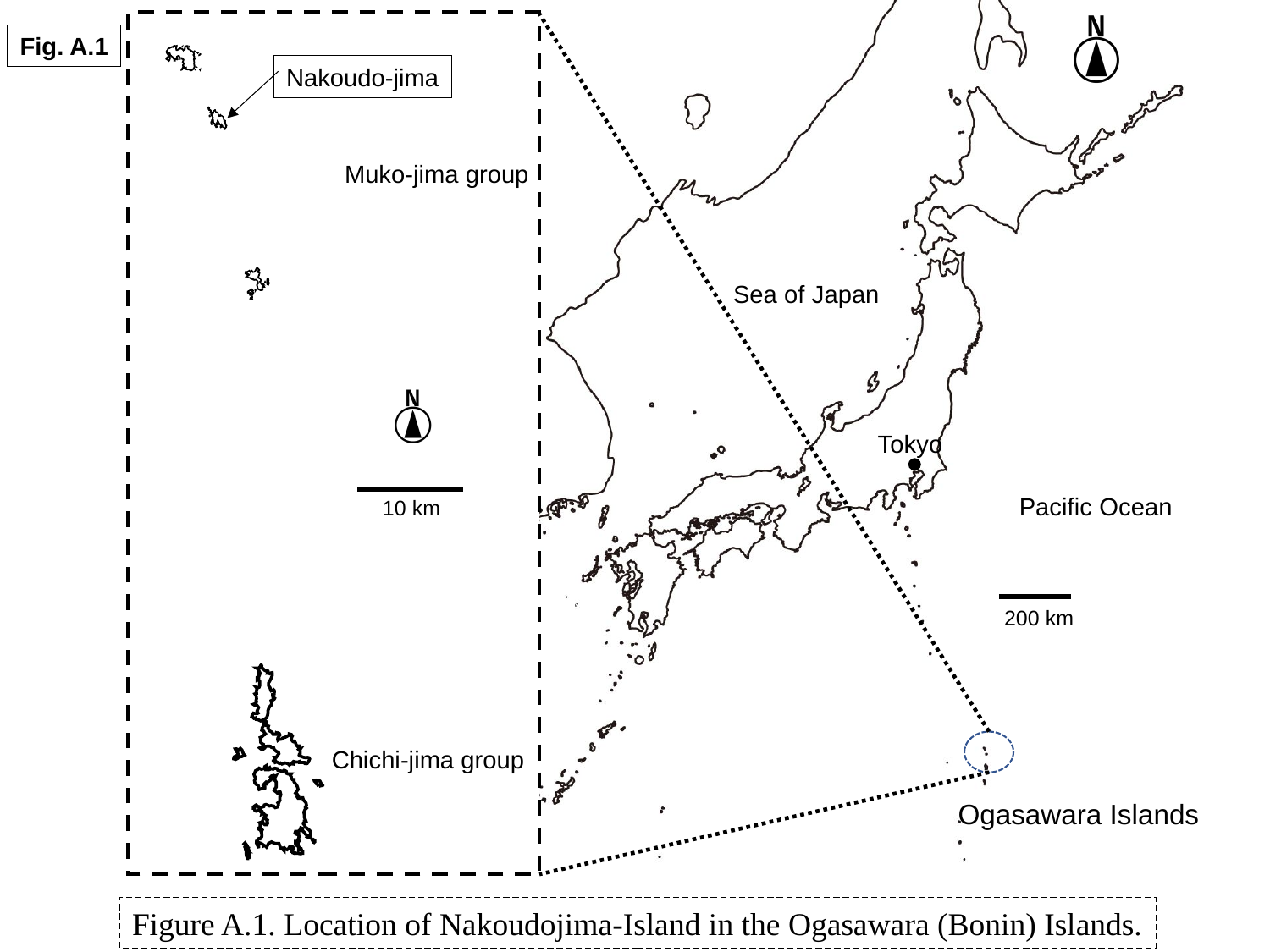

Fig. A.1
Nakoudo-jima
Muko-jima group
Sea of Japan
Tokyo
Pacific Ocean
10 km
200 km
Chichi-jima group
Ogasawara Islands
Figure A.1. Location of Nakoudojima-Island in the Ogasawara (Bonin) Islands.

## Slide 2
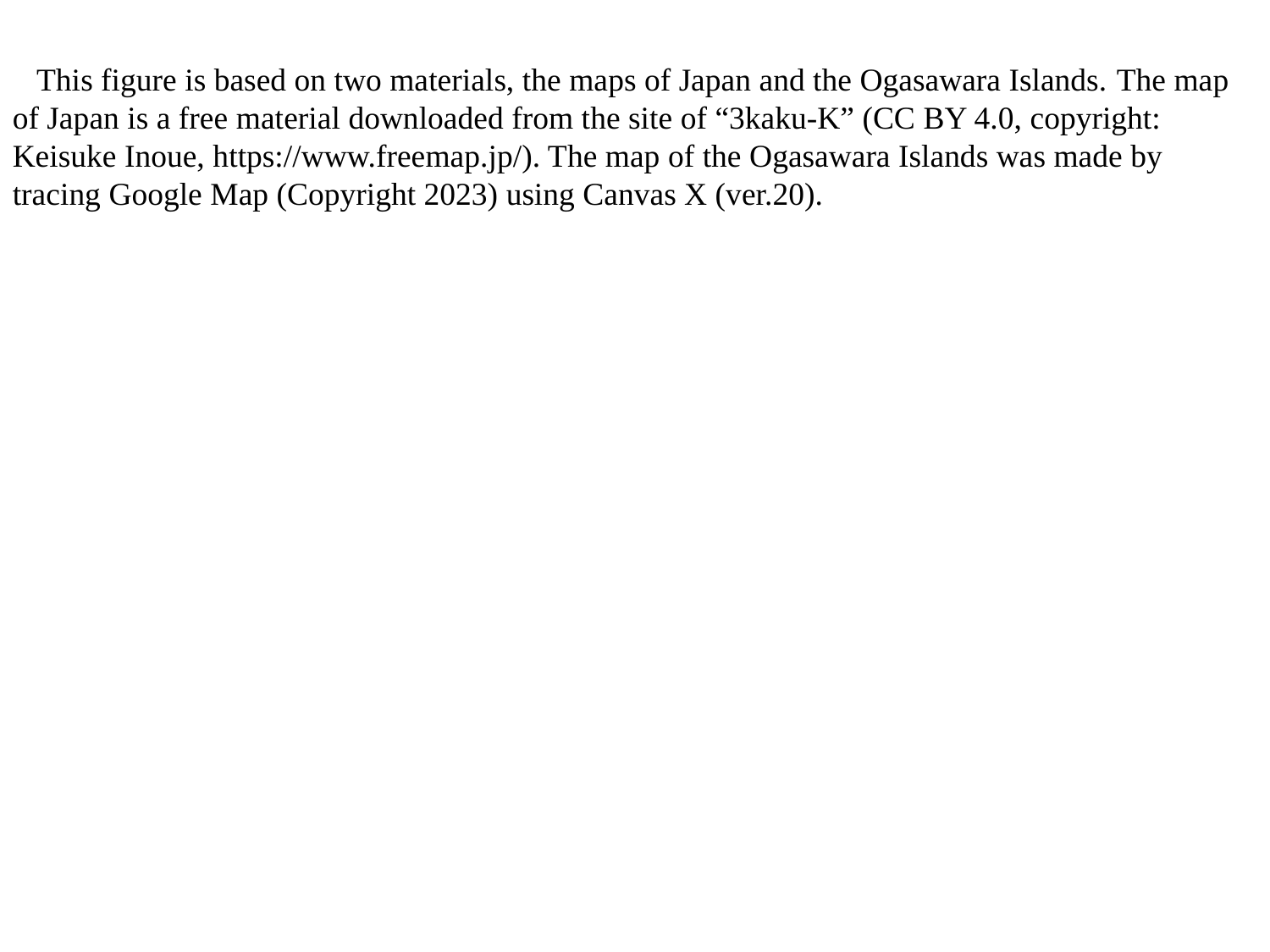

This figure is based on two materials, the maps of Japan and the Ogasawara Islands. The map of Japan is a free material downloaded from the site of “3kaku-K” (CC BY 4.0, copyright: Keisuke Inoue, https://www.freemap.jp/). The map of the Ogasawara Islands was made by tracing Google Map (Copyright 2023) using Canvas X (ver.20).

## Slide 3
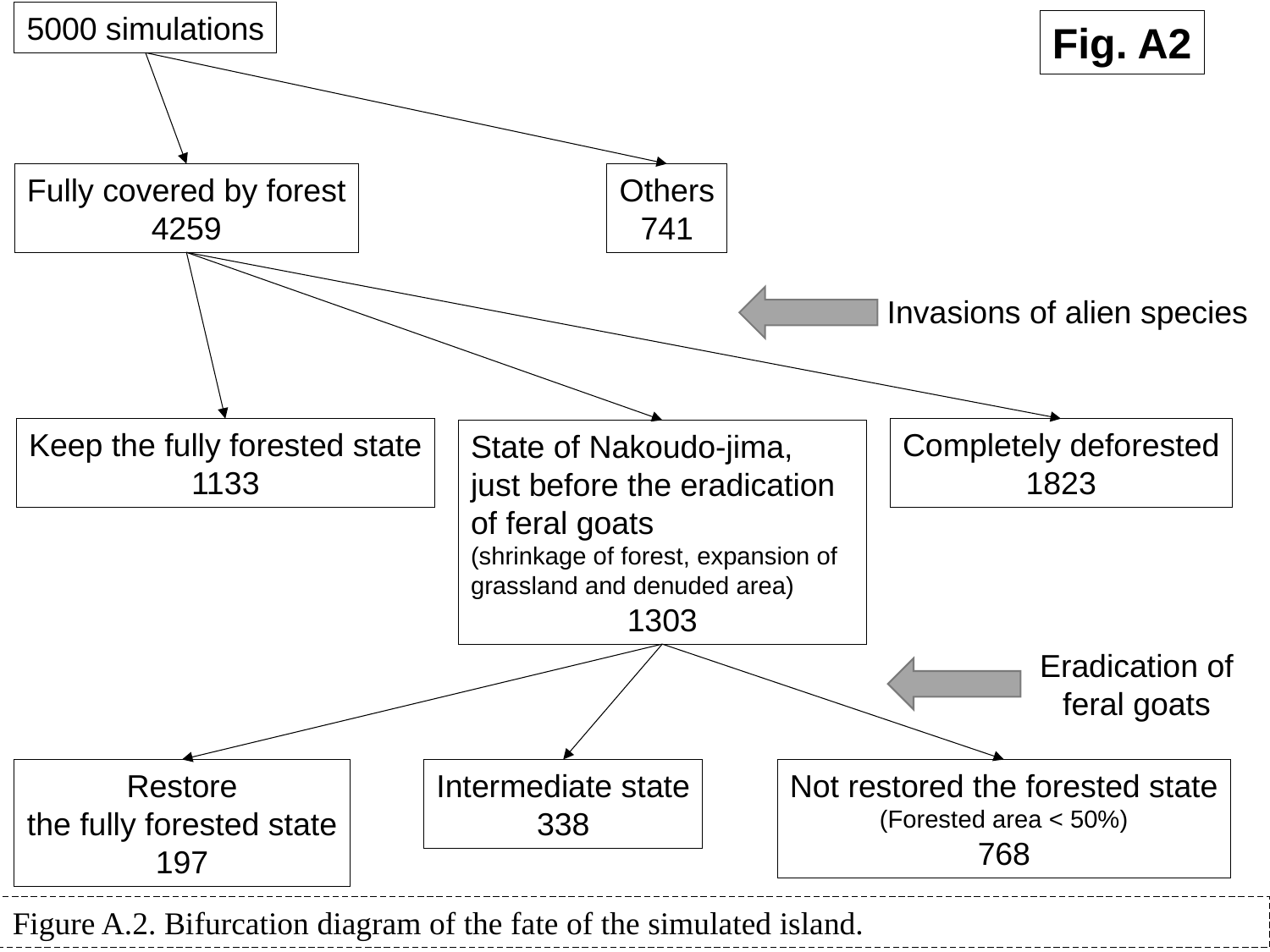

5000 simulations
Fig. A2
Fully covered by forest
4259
Others
741
Invasions of alien species
Completely deforested
1823
Keep the fully forested state
1133
State of Nakoudo-jima,
just before the eradication of feral goats
(shrinkage of forest, expansion of grassland and denuded area)
1303
Eradication of
feral goats
Restore
the fully forested state
197
Intermediate state
338
Not restored the forested state
(Forested area < 50%)
768
Figure A.2. Bifurcation diagram of the fate of the simulated island.

## Slide 4
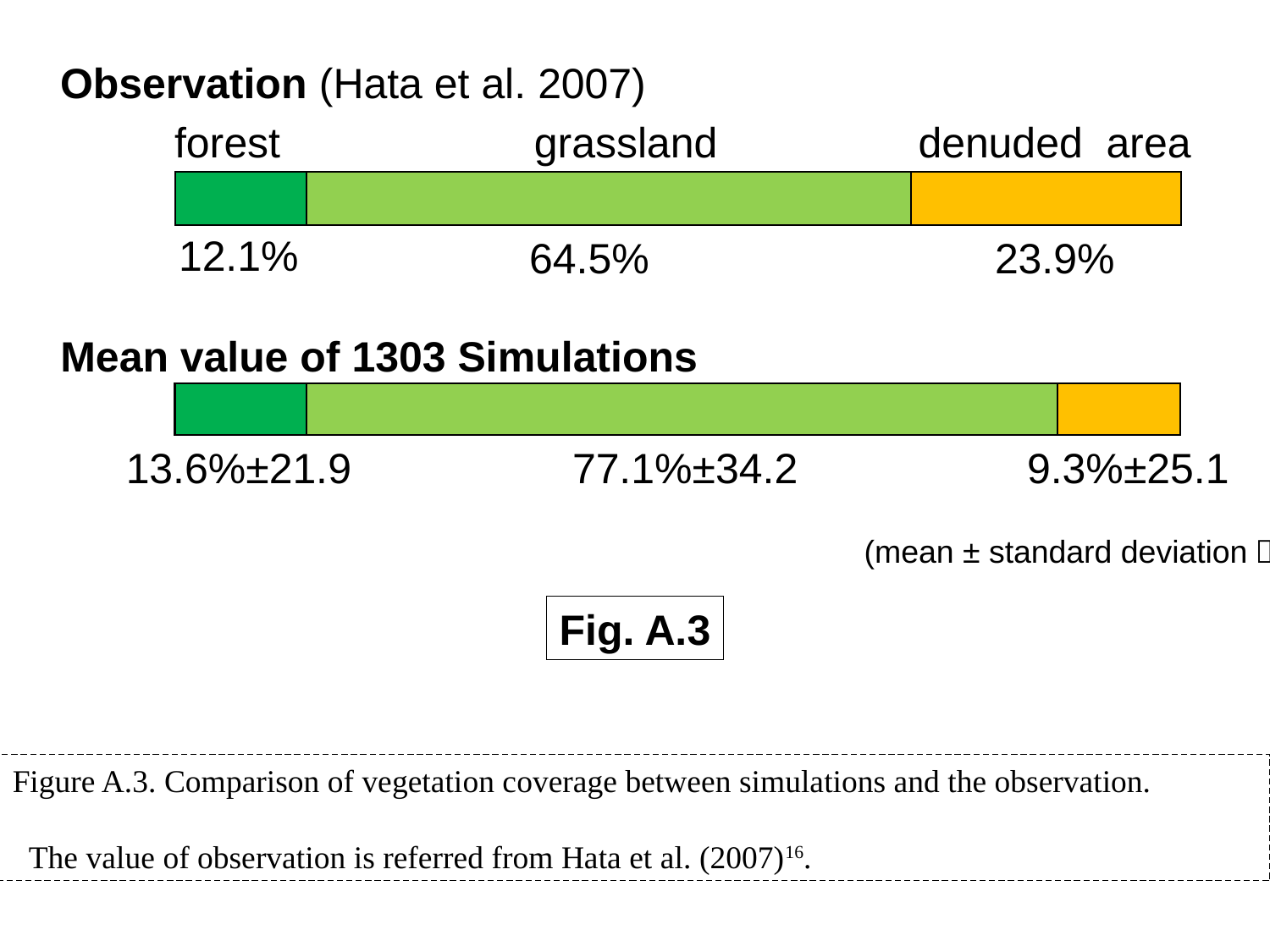

Observation (Hata et al. 2007)
denuded area
forest
grassland
12.1%
64.5%
23.9%
Mean value of 1303 Simulations
13.6%±21.9
77.1%±34.2
9.3%±25.1
(mean ± standard deviation）
Fig. A.3
Figure A.3. Comparison of vegetation coverage between simulations and the observation.
 The value of observation is referred from Hata et al. (2007)16.

## Slide 5
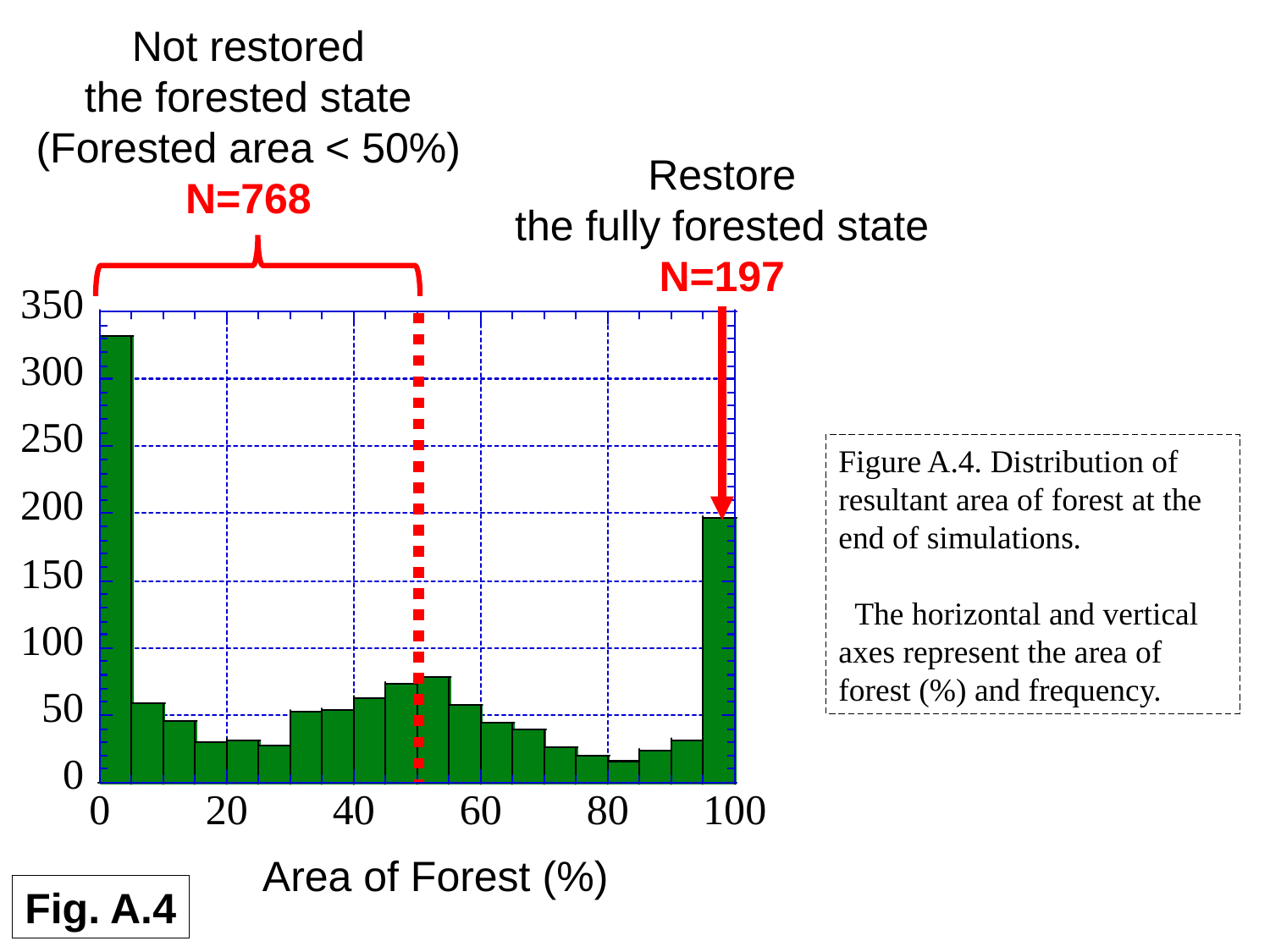

Not restored
the forested state
(Forested area < 50%)
N=768
Restore
the fully forested state
N=197
Figure A.4. Distribution of resultant area of forest at the end of simulations.
 The horizontal and vertical axes represent the area of forest (%) and frequency.
Area of Forest (%)
Fig. A.4

## Slide 6
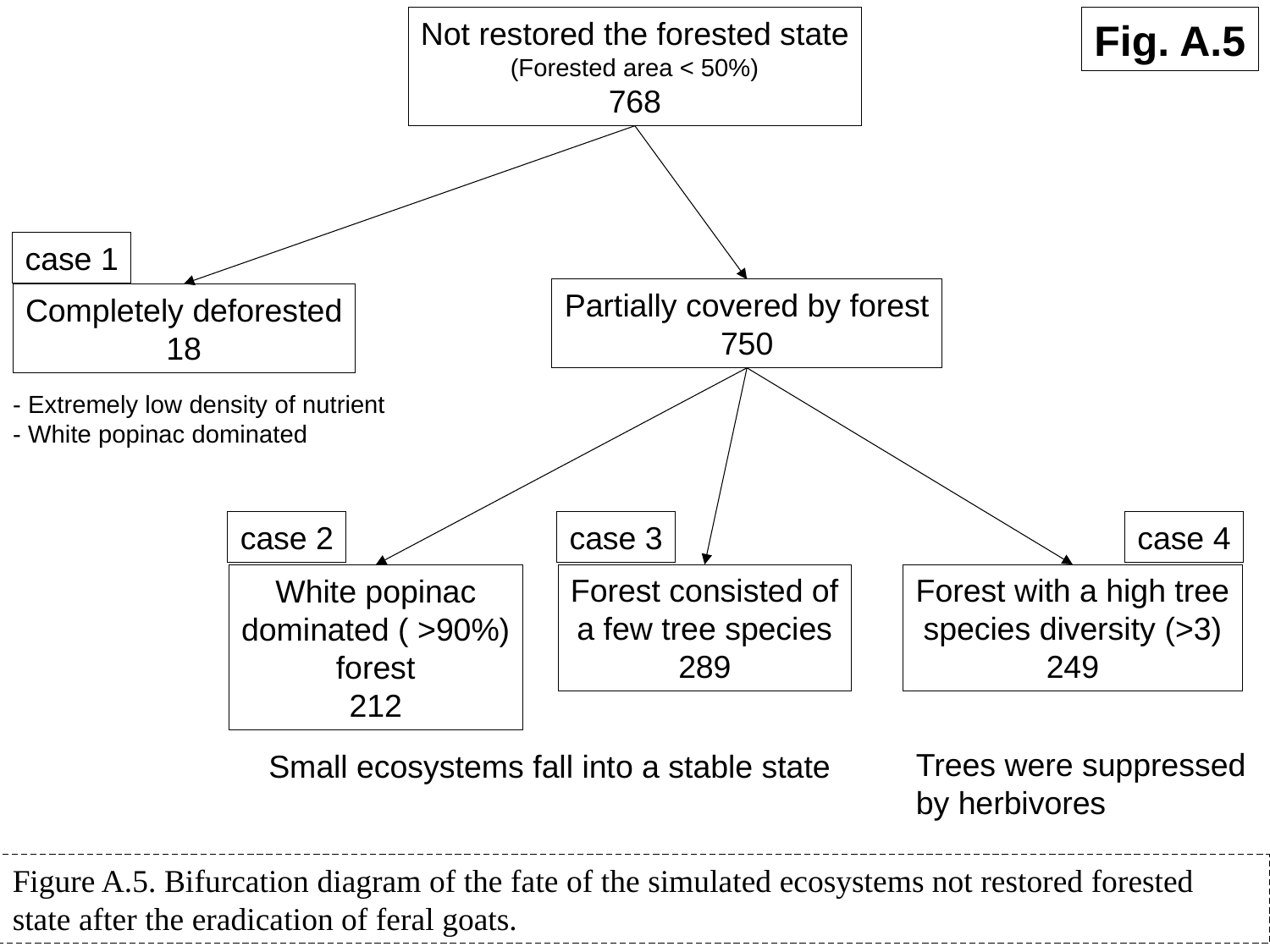

Fig. A.5
Not restored the forested state
(Forested area < 50%)
768
case 1
Partially covered by forest
750
Completely deforested
18
- Extremely low density of nutrient
- White popinac dominated
case 2
case 3
case 4
Forest consisted of
a few tree species
289
Forest with a high tree
species diversity (>3)
249
White popinac
dominated ( >90%)
forest
212
Trees were suppressed
by herbivores
Small ecosystems fall into a stable state
Figure A.5. Bifurcation diagram of the fate of the simulated ecosystems not restored forested state after the eradication of feral goats.

## Slide 7
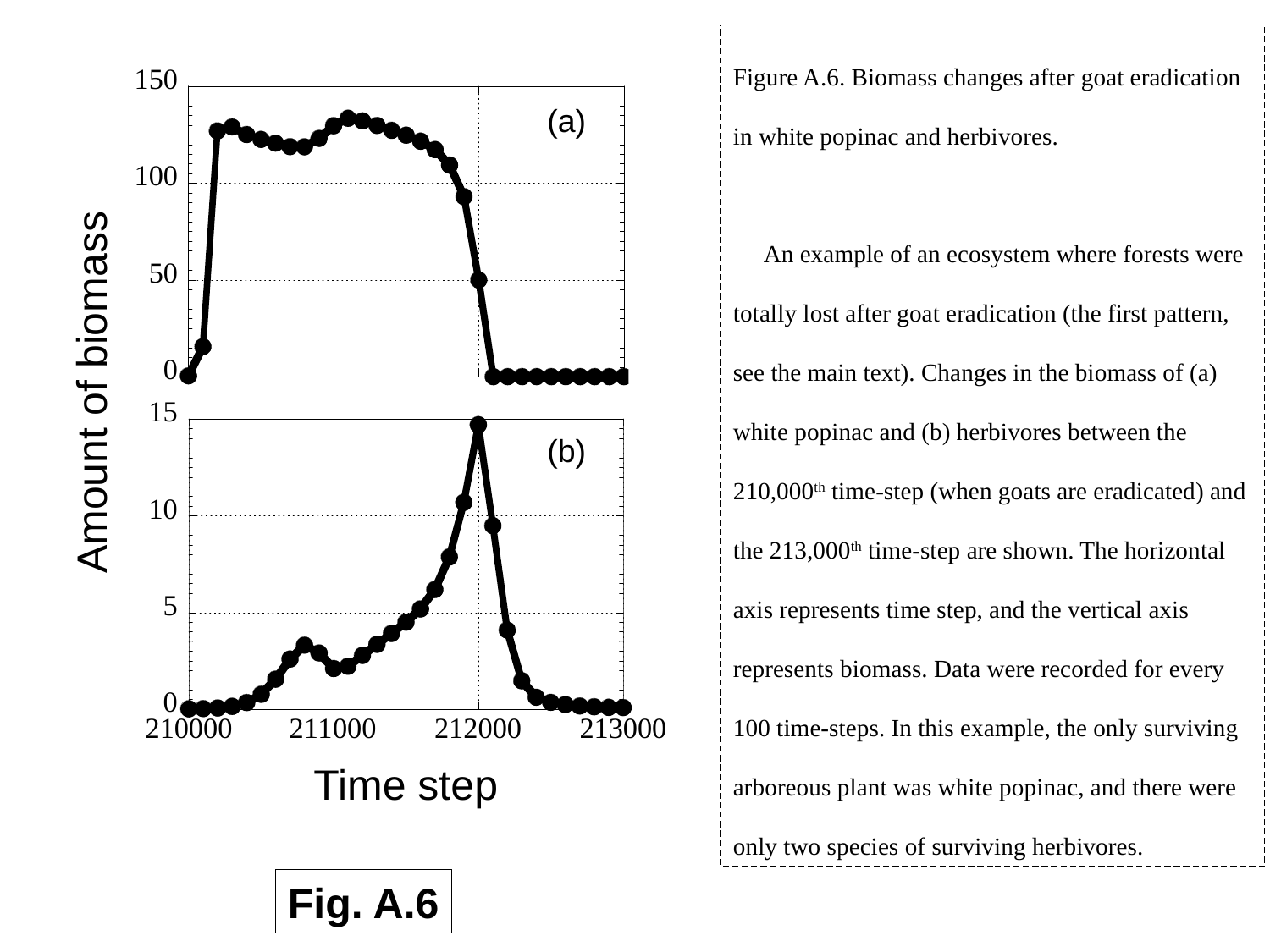

Figure A.6. Biomass changes after goat eradication in white popinac and herbivores.
　An example of an ecosystem where forests were totally lost after goat eradication (the first pattern, see the main text). Changes in the biomass of (a) white popinac and (b) herbivores between the 210,000th time-step (when goats are eradicated) and the 213,000th time-step are shown. The horizontal axis represents time step, and the vertical axis represents biomass. Data were recorded for every 100 time-steps. In this example, the only surviving arboreous plant was white popinac, and there were only two species of surviving herbivores.
(a)
Amount of biomass
(b)
Time step
Fig. A.6

## Slide 8
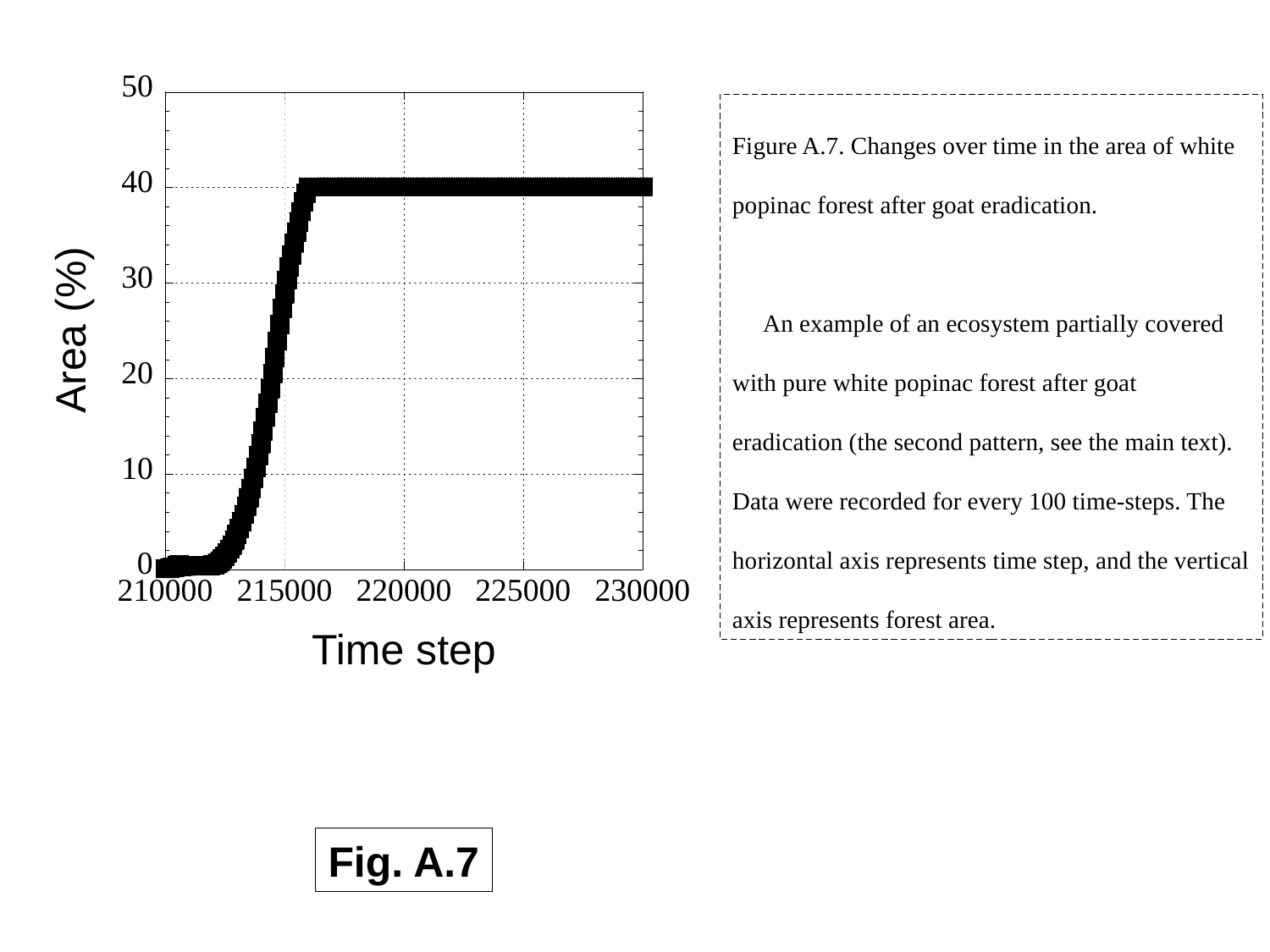

Figure A.7. Changes over time in the area of white popinac forest after goat eradication.
　An example of an ecosystem partially covered with pure white popinac forest after goat eradication (the second pattern, see the main text). Data were recorded for every 100 time-steps. The horizontal axis represents time step, and the vertical axis represents forest area.
Area (%)
Time step
Fig. A.7

## Slide 9
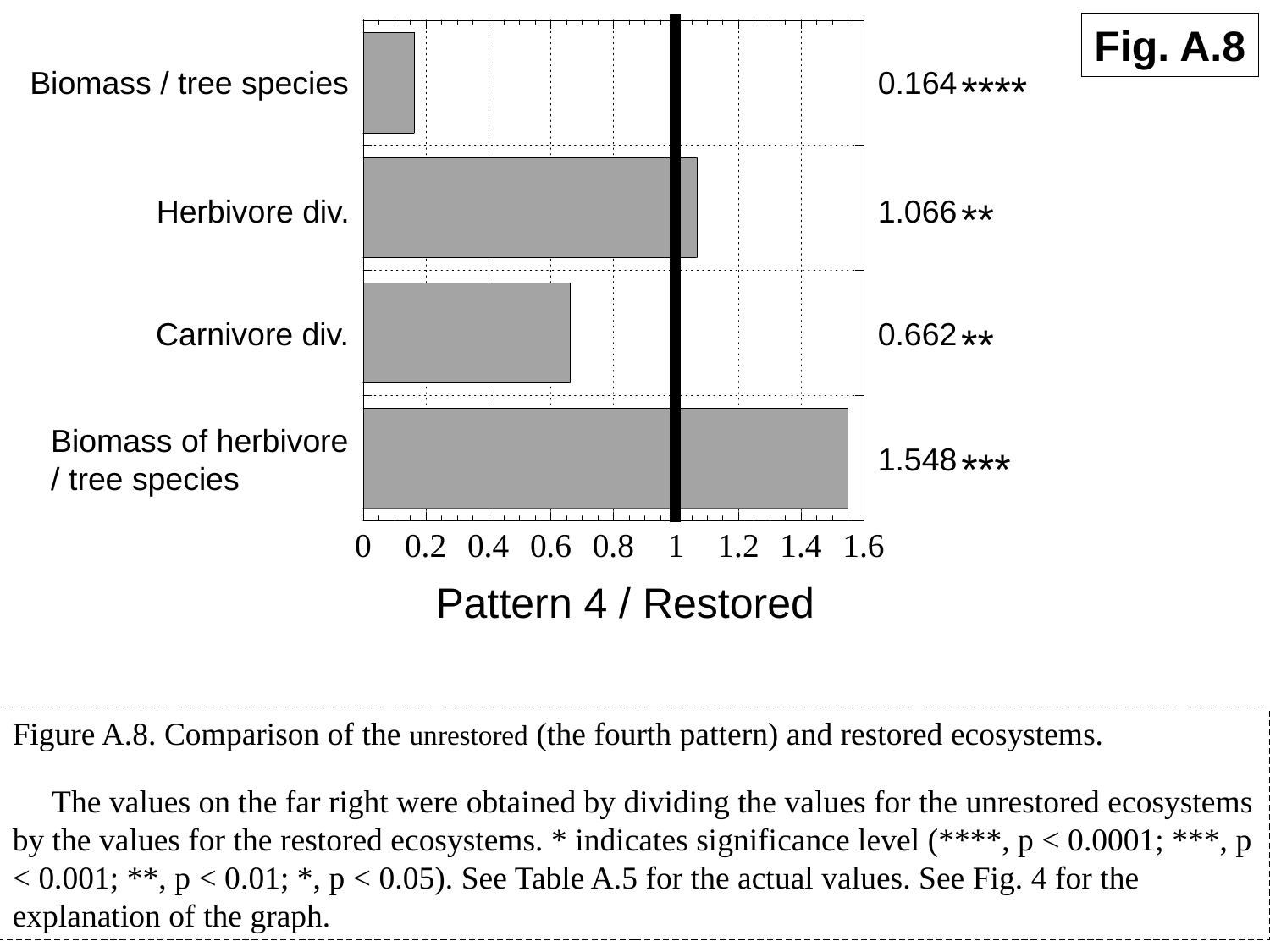

Fig. A.8
Biomass / tree species
0.164
****
Herbivore div.
1.066
**
Carnivore div.
0.662
**
Biomass of herbivore
/ tree species
1.548
***
Pattern 4 / Restored
Figure A.8. Comparison of the unrestored (the fourth pattern) and restored ecosystems.
　The values on the far right were obtained by dividing the values for the unrestored ecosystems by the values for the restored ecosystems. * indicates significance level (****, p < 0.0001; ***, p < 0.001; **, p < 0.01; *, p < 0.05). See Table A.5 for the actual values. See Fig. 4 for the explanation of the graph.

## Slide 10
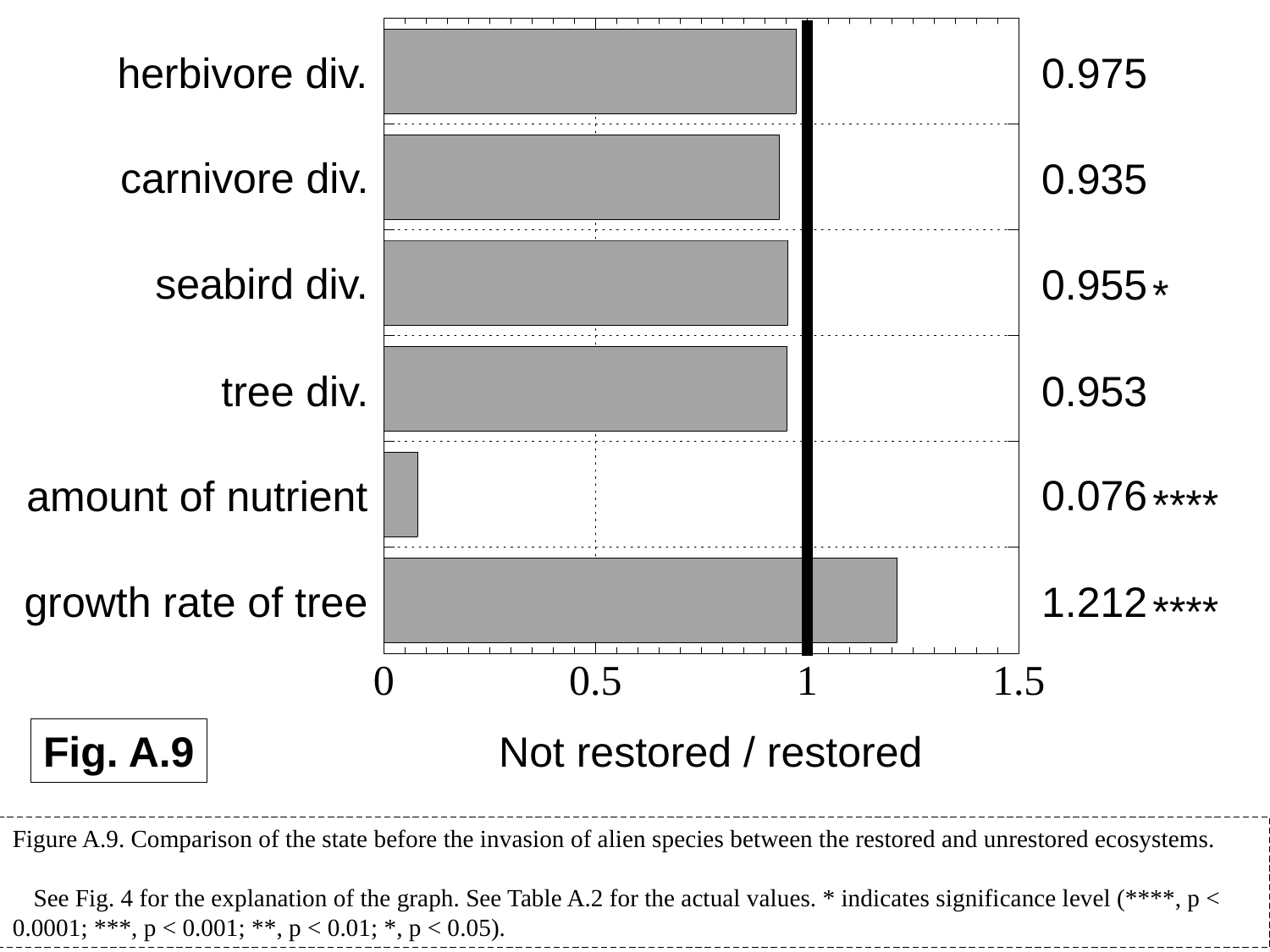

0.975
herbivore div.
carnivore div.
0.935
seabird div.
0.955
*
tree div.
0.953
0.076
amount of nutrient
****
growth rate of tree
1.212
****
Fig. A.9
Not restored / restored
Figure A.9. Comparison of the state before the invasion of alien species between the restored and unrestored ecosystems.
See Fig. 4 for the explanation of the graph. See Table A.2 for the actual values. * indicates significance level (****, p < 0.0001; ***, p < 0.001; **, p < 0.01; *, p < 0.05).

## Slide 11
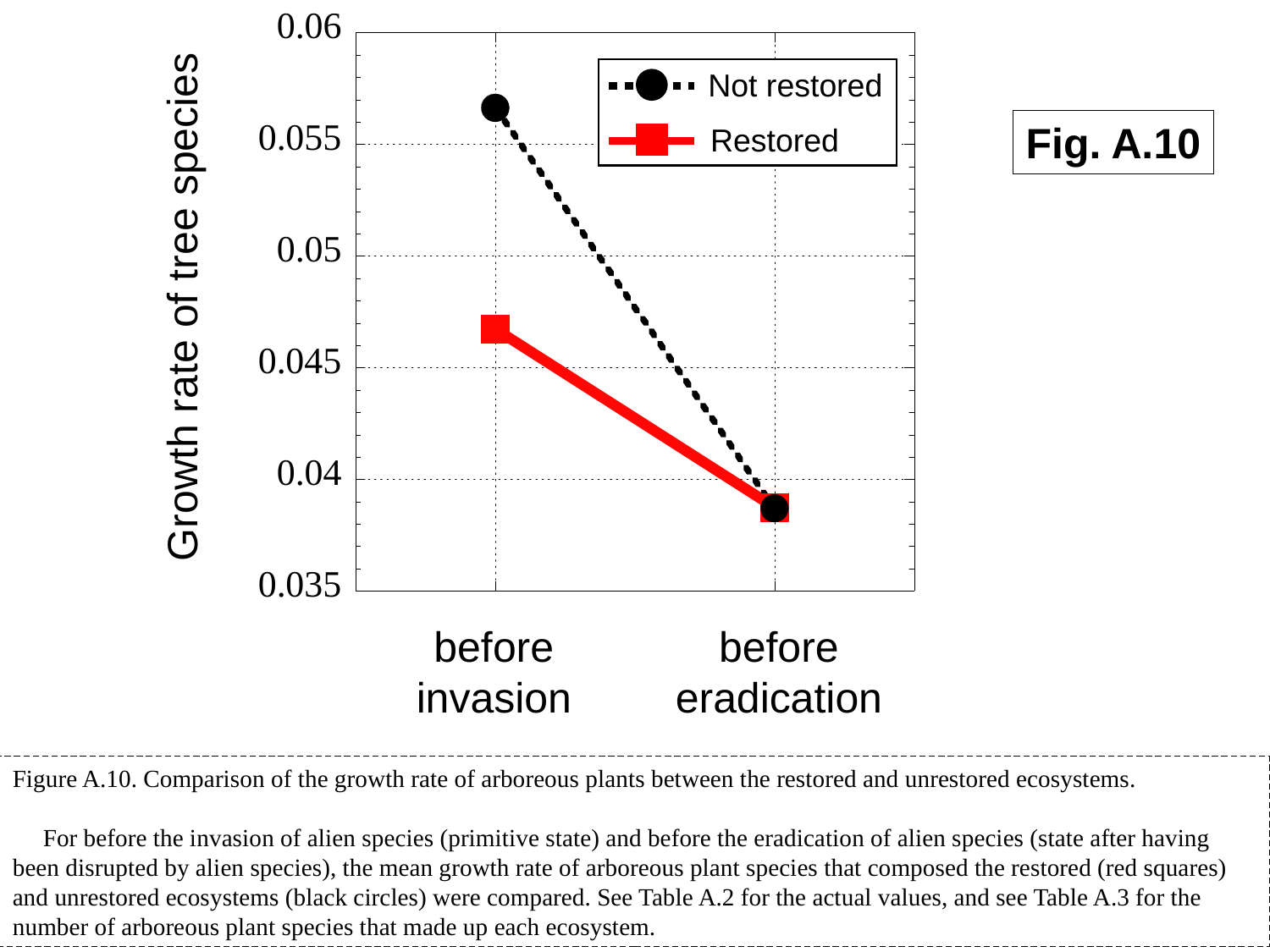

Not restored
Restored
Fig. A.10
Growth rate of tree species
before
invasion
before
eradication
Figure A.10. Comparison of the growth rate of arboreous plants between the restored and unrestored ecosystems.
　For before the invasion of alien species (primitive state) and before the eradication of alien species (state after having been disrupted by alien species), the mean growth rate of arboreous plant species that composed the restored (red squares) and unrestored ecosystems (black circles) were compared. See Table A.2 for the actual values, and see Table A.3 for the number of arboreous plant species that made up each ecosystem.

## Slide 12
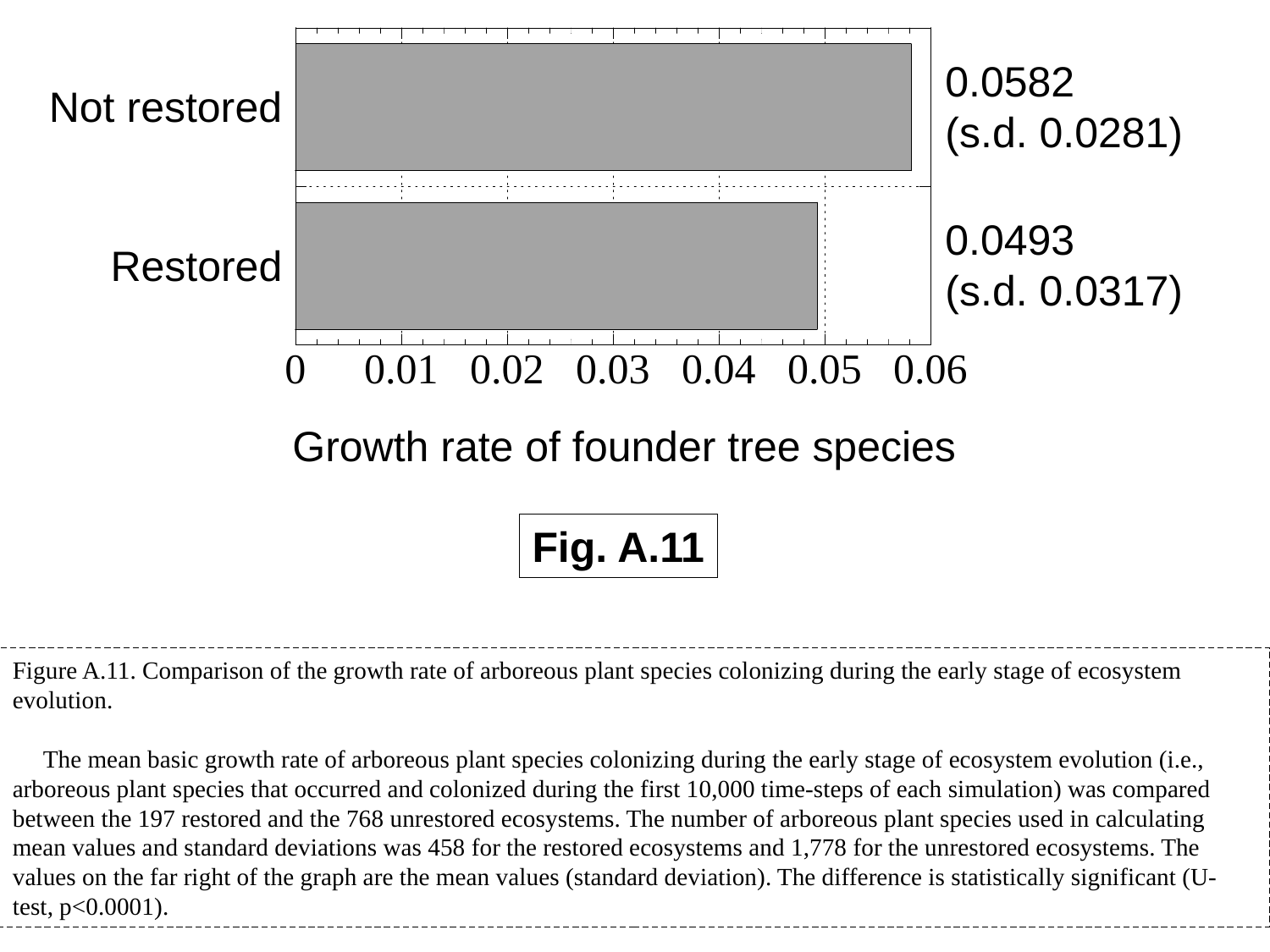

0.0582
(s.d. 0.0281)
Not restored
0.0493
(s.d. 0.0317)
Restored
Growth rate of founder tree species
Fig. A.11
Figure A.11. Comparison of the growth rate of arboreous plant species colonizing during the early stage of ecosystem evolution.
　The mean basic growth rate of arboreous plant species colonizing during the early stage of ecosystem evolution (i.e., arboreous plant species that occurred and colonized during the first 10,000 time-steps of each simulation) was compared between the 197 restored and the 768 unrestored ecosystems. The number of arboreous plant species used in calculating mean values and standard deviations was 458 for the restored ecosystems and 1,778 for the unrestored ecosystems. The values on the far right of the graph are the mean values (standard deviation). The difference is statistically significant (U-test, p<0.0001).

## Slide 13
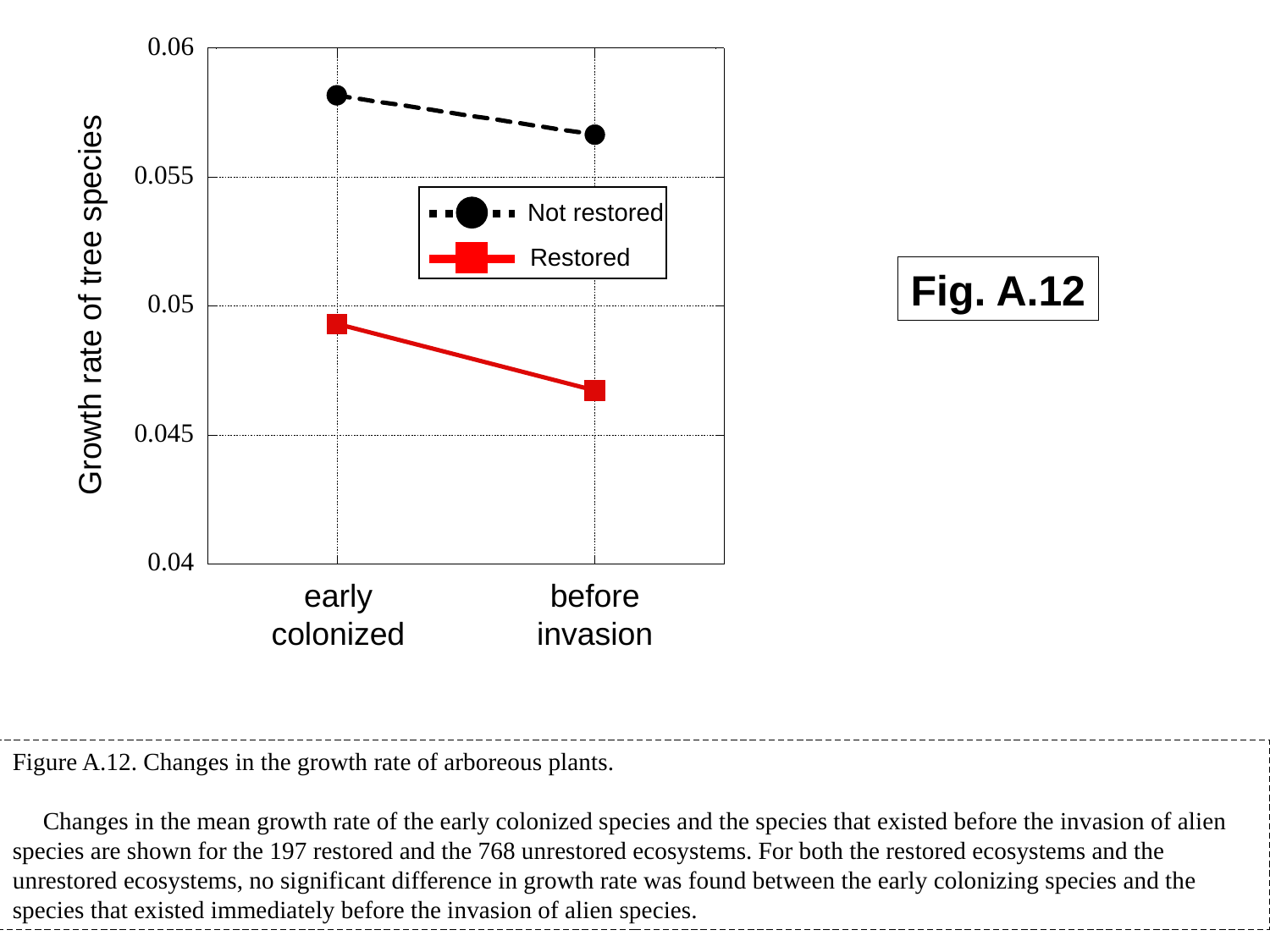

Not restored
Restored
Fig. A.12
Growth rate of tree species
before
invasion
early
colonized
Figure A.12. Changes in the growth rate of arboreous plants.
　Changes in the mean growth rate of the early colonized species and the species that existed before the invasion of alien species are shown for the 197 restored and the 768 unrestored ecosystems. For both the restored ecosystems and the unrestored ecosystems, no significant difference in growth rate was found between the early colonizing species and the species that existed immediately before the invasion of alien species.

## Slide 14
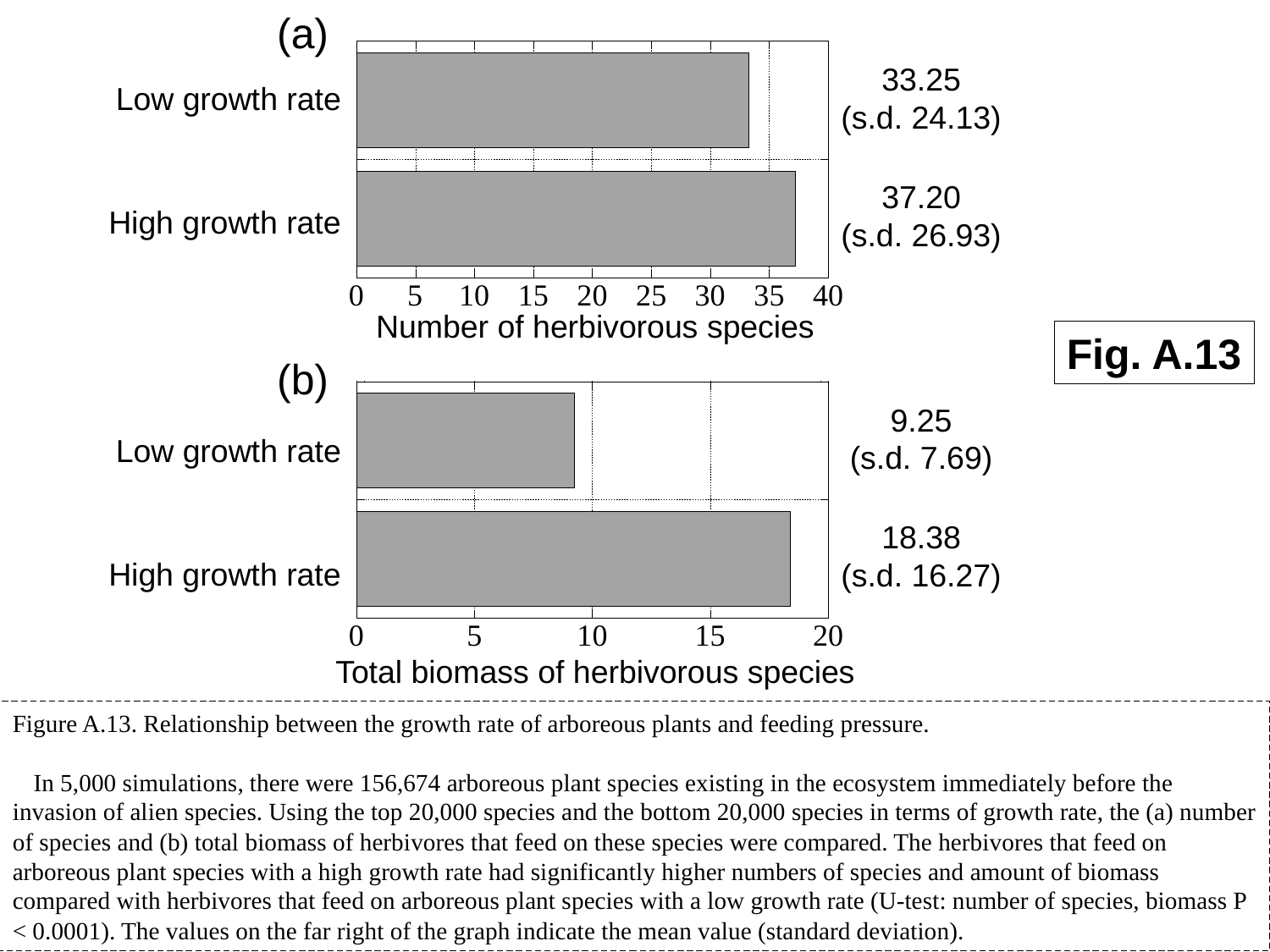

(a)
33.25
(s.d. 24.13)
Low growth rate
37.20
(s.d. 26.93)
High growth rate
Number of herbivorous species
Fig. A.13
(b)
9.25
(s.d. 7.69)
Low growth rate
18.38
(s.d. 16.27)
High growth rate
Total biomass of herbivorous species
Figure A.13. Relationship between the growth rate of arboreous plants and feeding pressure.
In 5,000 simulations, there were 156,674 arboreous plant species existing in the ecosystem immediately before the invasion of alien species. Using the top 20,000 species and the bottom 20,000 species in terms of growth rate, the (a) number of species and (b) total biomass of herbivores that feed on these species were compared. The herbivores that feed on arboreous plant species with a high growth rate had significantly higher numbers of species and amount of biomass compared with herbivores that feed on arboreous plant species with a low growth rate (U-test: number of species, biomass P < 0.0001). The values on the far right of the graph indicate the mean value (standard deviation).

## Slide 15
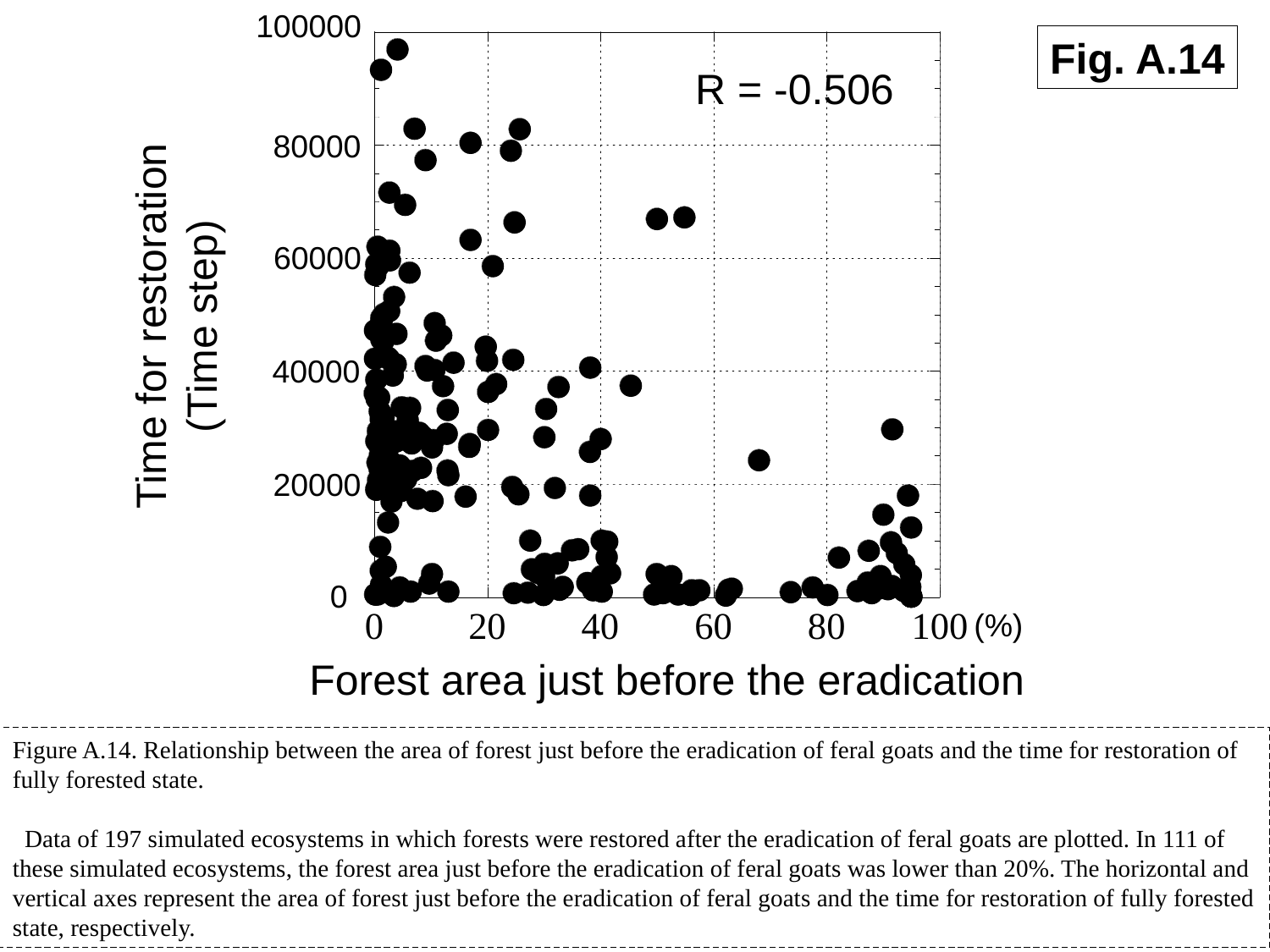

100000
Fig. A.14
R = -0.506
80000
60000
Time for restoration
(Time step)
40000
20000
0
(%)
Forest area just before the eradication
Figure A.14. Relationship between the area of forest just before the eradication of feral goats and the time for restoration of fully forested state.
 Data of 197 simulated ecosystems in which forests were restored after the eradication of feral goats are plotted. In 111 of these simulated ecosystems, the forest area just before the eradication of feral goats was lower than 20%. The horizontal and vertical axes represent the area of forest just before the eradication of feral goats and the time for restoration of fully forested state, respectively.

## Slide 16
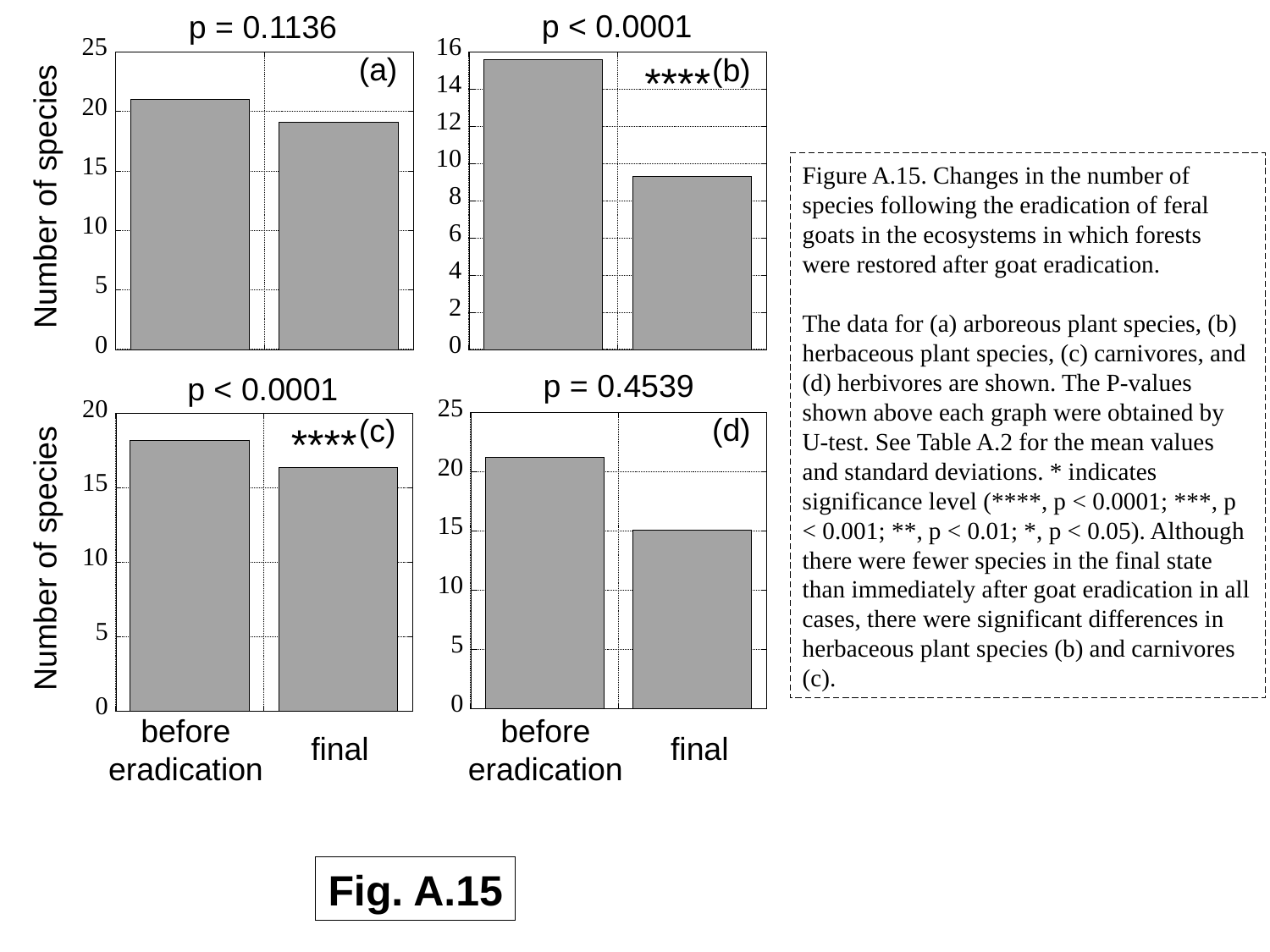

p < 0.0001
p = 0.1136
(a)
(b)
****
Figure A.15. Changes in the number of species following the eradication of feral goats in the ecosystems in which forests were restored after goat eradication.
The data for (a) arboreous plant species, (b) herbaceous plant species, (c) carnivores, and (d) herbivores are shown. The P-values shown above each graph were obtained by U-test. See Table A.2 for the mean values and standard deviations. * indicates significance level (****, p < 0.0001; ***, p < 0.001; **, p < 0.01; *, p < 0.05). Although there were fewer species in the final state than immediately after goat eradication in all cases, there were significant differences in herbaceous plant species (b) and carnivores (c).
Number of species
p = 0.4539
p < 0.0001
(d)
(c)
****
Number of species
before
eradication
before
eradication
final
final
Fig. A.15

## Slide 17
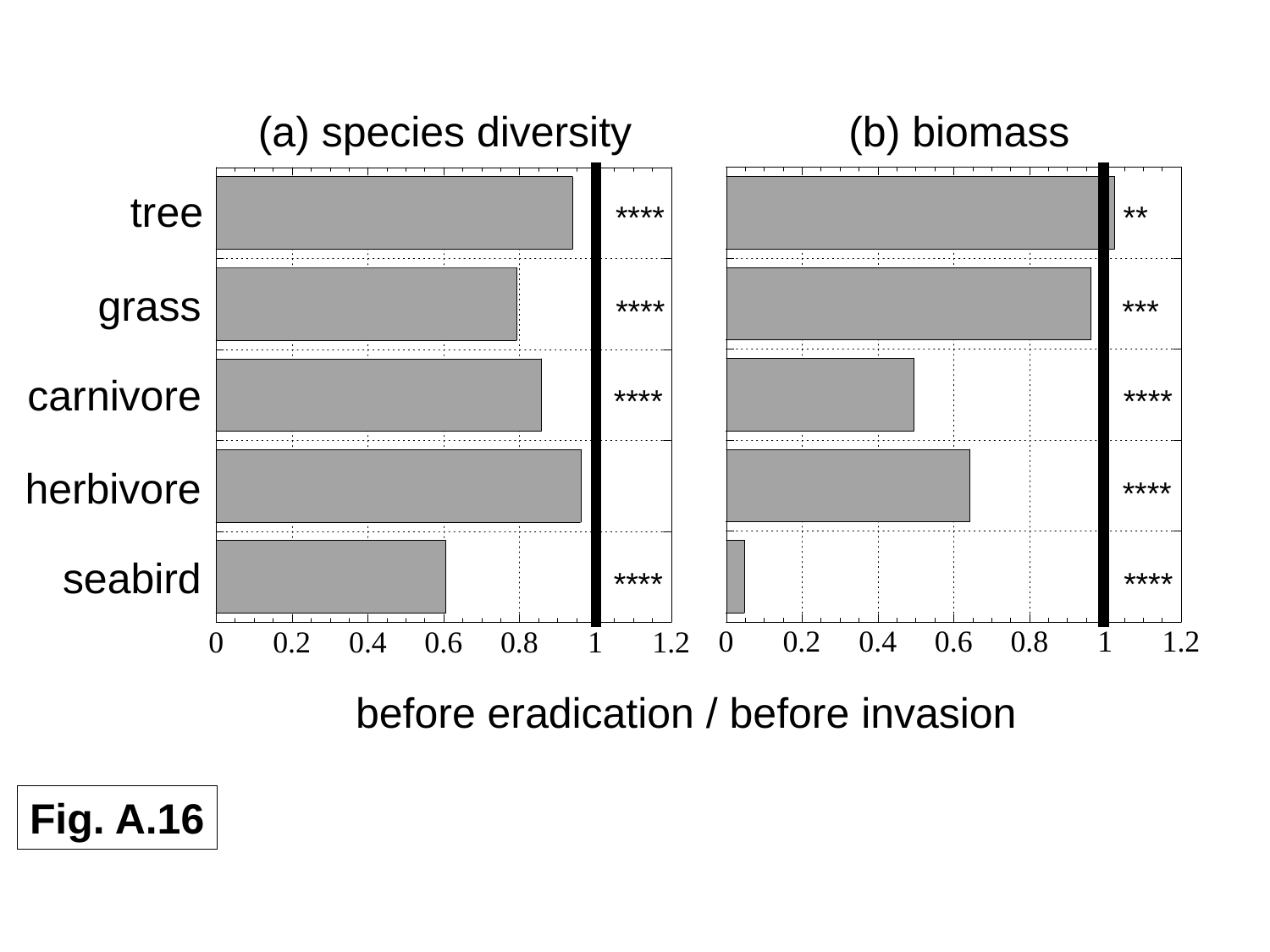

(a) species diversity
(b) biomass
tree
****
**
grass
****
***
carnivore
****
****
herbivore
****
seabird
****
****
before eradication / before invasion
Fig. A.16

## Slide 18
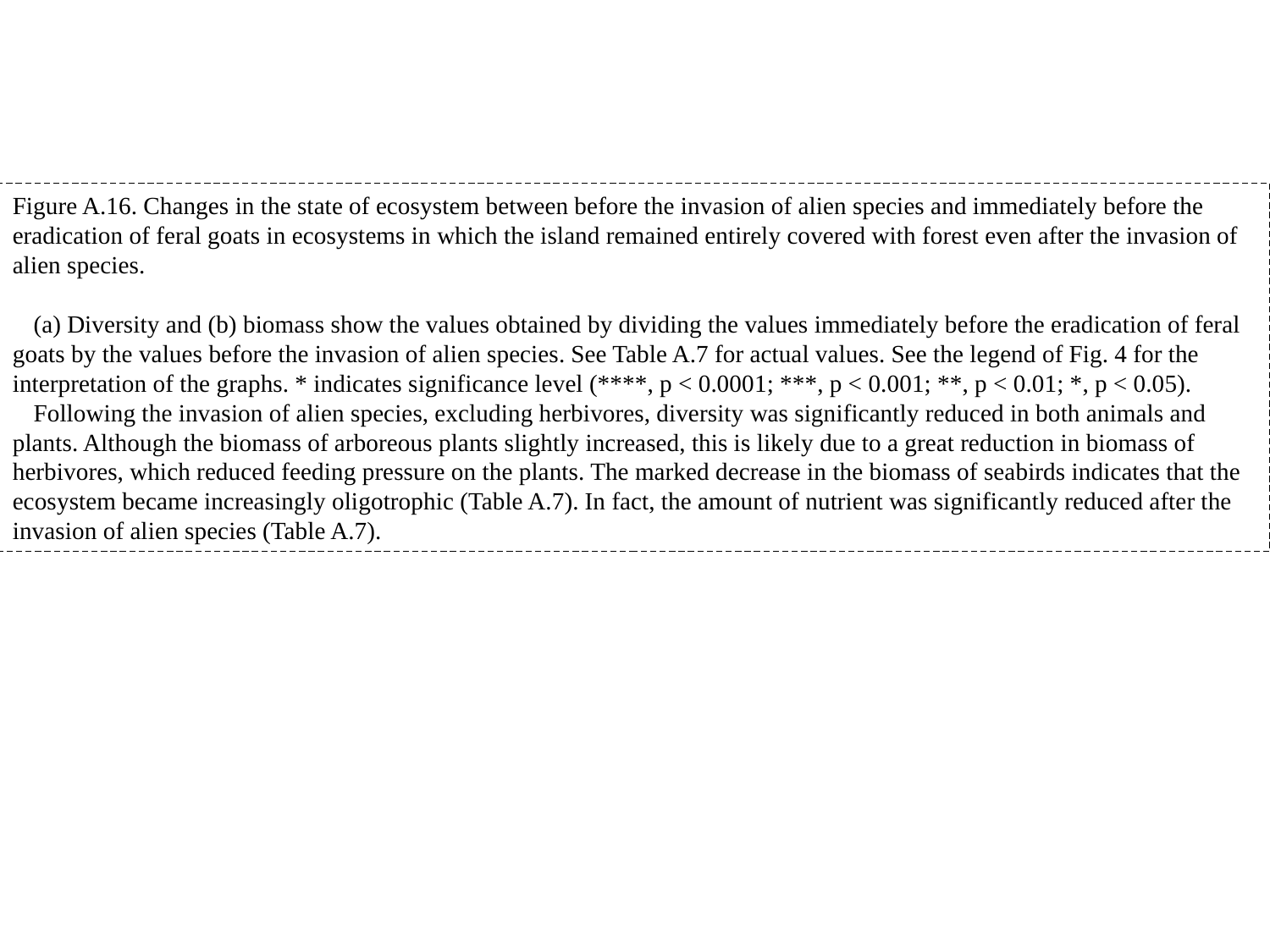

Figure A.16. Changes in the state of ecosystem between before the invasion of alien species and immediately before the eradication of feral goats in ecosystems in which the island remained entirely covered with forest even after the invasion of alien species.
(a) Diversity and (b) biomass show the values obtained by dividing the values immediately before the eradication of feral goats by the values before the invasion of alien species. See Table A.7 for actual values. See the legend of Fig. 4 for the interpretation of the graphs. * indicates significance level (****, p < 0.0001; ***, p < 0.001; **, p < 0.01; *, p < 0.05).
Following the invasion of alien species, excluding herbivores, diversity was significantly reduced in both animals and plants. Although the biomass of arboreous plants slightly increased, this is likely due to a great reduction in biomass of herbivores, which reduced feeding pressure on the plants. The marked decrease in the biomass of seabirds indicates that the ecosystem became increasingly oligotrophic (Table A.7). In fact, the amount of nutrient was significantly reduced after the invasion of alien species (Table A.7).
